# Succinate-driven virulence enhancement in hypervirulent *Klebsiella pneumoniae* via DcuSR two-component system

**DOI:** 10.1128/spectrum.01453-25

**Published:** 2025-11-13

**Authors:** Guosheng Zhong, Xiaoya Yang, Ruting Deng, Jianming Zeng, Jieying Pu, Cong Shen, Siyu Zhao, Tingting Yang, Huanliang Liu, Mingyong Luo, Shibing Li, Cha Chen, Bin Huang

**Affiliations:** 1 Department of Clinical Laboratory, The Sixth Affiliated Hospital, Sun Yat-sen University 26469 , Guangzhou, China; 2 Ministry of Education, Key Laboratory of Human Microbiome and Chronic Diseases (Sun Yat-sen University) 26469 , Guangzhou, China; 3 Biomedical Innovation Center, The Sixth Affiliated Hospital, Sun Yat-sen University 26469 , Guangzhou, China; 4 Department of Clinical Laboratory, Guangdong Women and Children Hospital 90405 , Guangzhou, China; 5 Women and Children’s Hospital, Southern University of Science and Technology, Guangzhou, China; 6 Department of Laboratory Medicine, The First Affiliated Hospital, Sun Yat-sen University 199192 , Guangzhou, China; 7 Department of Laboratory Medicine, The Second Affiliated Hospital, Guangzhou University of Chinese Medicine 47879, Guangzhou, China; University of Guelph College of Biological Science, Guelph, Ontario, Canada

**Keywords:** succinate, hvKP, DcuSR, virulence, ATP synthesis

## Abstract

Hypervirulent *Klebsiella pneumoniae* (hvKP) primarily colonizes the mammalian gastrointestinal tract, and it is prone to causing invasive infections under specific conditions. To establish infection, hvKP must compete with the resident gut microbiota for essential nutrients. This study focuses on the C_4_-dicarboxylate (C_4_-DC) succinate, which has been reported to accumulate in the gut under specific pathological conditions. We demonstrate that hvKP utilizes succinate as a signaling molecule to enhance virulence gene expression. Specifically, extracellular succinate activates type VI secretion system gene expression via the DcuSR two-component system (TCS), thereby increasing cytotoxicity toward intestinal epithelial cells and enhancing competitiveness against commensal *Escherichia coli*. Additionally, succinate facilitates the expression of type III fimbriae via the DcuSR TCS, promoting hvKP adherence to intestinal epithelial cells. Beyond its signaling role, succinate functions as a metabolic substrate and contributes to adenosine 5′-triphosphate (ATP) synthesis, potentially through C_4_-dicarboxylic acid transporters DctA or DcuB to boost energy synthesis. This study focuses on elucidating the impact of succinate in regulating hvKP virulence, with particular attention to the potential role of the DcuSR TCS in hvKP pathogenesis.

**IMPORTANCE:**

Succinate, a C_4_-DC, is produced by the host and the gut microbiota and can accumulate in the intestinal environment under various pathological conditions, such as diabetes and inflammatory bowel disease. It acts as a pivotal regulator of virulence traits and metabolic pathways in Enterobacteriaceae. Our findings highlight the significant impact of succinate on hvKP pathogenesis: (i) functioning through the DcuSR TCS to activate virulence programs and (ii) serving as a metabolic substrate that fuels bioenergetic adaptation through ATP synthesis.

## INTRODUCTION

Hypervirulent *Klebsiella pneumoniae* (hvKP) is an opportunistic pathogen responsible for severe community-acquired infections (1). Unlike classical *Klebsiella pneumoniae*, hvKP is characterized by high virulence, significant pathogenic potential, high mortality rates, and poor clinical prognosis, posing a major challenge in clinical management (2). hvKP demonstrates intestinal colonization capacity and may cause invasive infections, including liver abscess, lung abscess, meningitis, urinary tract infections, and endophthalmitis, collectively termed invasive *Klebsiella pneumoniae* liver abscess syndrome (3).

The strong invasive potential of hvKP is attributed to its virulence factors (4, 5). Key virulence determinants, including the capsule, siderophores, fimbriae, lipopolysaccharide, and the type VI secretion system (T6SS), contribute significantly to pathogenicity (3, 6, 7). T6SS has been widely reported in gram-negative pathogens, especially in Enterobacteriaceae (8). It is a molecular syringe and consists of three main subunits: membrane complex, baseplate complex, and tail tube/sheath complex (9). T6SS effectors, such as antibacterial proteins and toxins, can be injected into target cells, facilitating competition against commensal microbiota and host cells (10, 11). Notably, previous studies have implicated T6SS in invasive infection caused by *K. pneumoniae* (12, 13).

Successful colonization of the gastrointestinal tract is a prerequisite for hvKP to establish infection (14). However, the mammalian gut harbors a dense and diverse community of commensal microbiota that confer “colonization resistance,” a protective mechanism that inhibits the establishment of invasive pathogens (15, 16). hvKP must overcome the colonization resistance imposed by the gut microbiota to successfully colonize the intestine and exert its pathogenic effects (17). The mechanism through which hvKP adapts to the gut environment and establishes infection remains poorly understood.

Recent studies indicate that intestinal pathogens can sense and utilize host- and microbiota-derived metabolites to enhance survival and colonization (18–21). Succinate, a C_4_-dicarboxylate (C_4_-DC), is a key intermediate in the mitochondrial tricarboxylic acid (TCA) cycle in host cells and is also produced by gut microbiota through dietary fiber fermentation (22, 23). Under normal physiological conditions, a dynamic equilibrium between succinate-producing bacteria (e.g., *Bacteroides* and *Prevotella* genera) and succinate-consuming bacteria (e.g., *Phascolarctobacterium succinatutens* and *Dialister succinatiphilus*) maintains low succinate concentrations in the intestine. However, pathological conditions such as diabetes, inflammatory bowel disease, obesity, and antibiotic overuse disrupt this balance, leading to succinate accumulation of up to 24 mM (24–26). The elevated succinate levels in diseased states create a favorable niche for intestinal pathogens by serving as both a nutrient source and a regulatory signal. Pathogenic bacteria primarily import succinate via C_4_-DC transporters, such as DctA and DcuB (27, 28). For instance, *Salmonella serovar* Typhimurium exploits microbiota-derived succinate as an energy source to drive the TCA cycle, enhancing colonization competitiveness (29). However, whether succinate contributes to hvKP energy metabolism remains unclear.

Beyond its metabolic role, succinate also functions as a signaling molecule that modulates bacterial virulence. *Salmonella* Typhimurium exploits succinate accumulated in macrophages due to the shift from oxidative phosphorylation to glycolysis, utilizing the succinate to upregulate the type III secretion system (T3SS) encoded by *Salmonella* pathogenicity island 2 (30). Similarly, succinate enhances the expression of the locus of enterocyte effacement (LEE) genes in enterohemorrhagic *Escherichia coli* (EHEC), promoting adhesion and colonization (31). Moreover, succinate-stressed *Pseudomonas aeruginosa* overexpresses extracellular polysaccharides and glyoxylate shunt components, facilitating biofilm formation (32). Likewise, succinate enhances *Clostridium difficile* biofilm formation, reinforcing its competitive advantage against the gut microbiota (33). The DcuSR two-component system (TCS), composed of the sensor kinase DcuS and the response regulator DcuR, plays an important role in sensing the presence of external C_4_-dicarboxylates, such as succinate (27, 34), fumarate (35, 36), L-malate (37), and L-aspartate (38), and induces C_4_-dicarboxylate metabolism genes. Notably, EHEC employs the DcuSR TCS to sense L-malate from both host and microbial sources, which subsequently, in addition to triggering fumarate respiration, also promotes elevated LEE gene expression and promotes colonization in the host (37). However, whether hvKP exploits succinate as a signaling molecule to regulate virulence gene expression—and whether DcuSR TCS mediates this process—remains largely unknown.

In this study, we reveal that succinate functions as a signaling molecule to enhance hvKP virulence expression. Specifically, we demonstrate that the DcuSR TCS plays an essential role in mediating succinate-dependent enhancement of key virulence factors, including T6SS and type III fimbriae. Deletion of *dcuS* or *dcuR* significantly attenuates virulence gene expression, reduces hvKP adherence to intestinal epithelial cells, and diminishes pathogenicity *in vitro*. Furthermore, our data indicate succinate enhances adenosine 5′-triphosphate (ATP) production in hvKP, potentially through DctA or DcuB transporters. These findings link succinate availability to both virulence and metabolic adaptation, offering a potential therapeutic avenue by targeting DcuSR TCS for the prevention and treatment of invasive hvKP infections.

## RESULTS

### Succinate enhances the virulence and pathogenicity of hvKP

**Fig 1:**
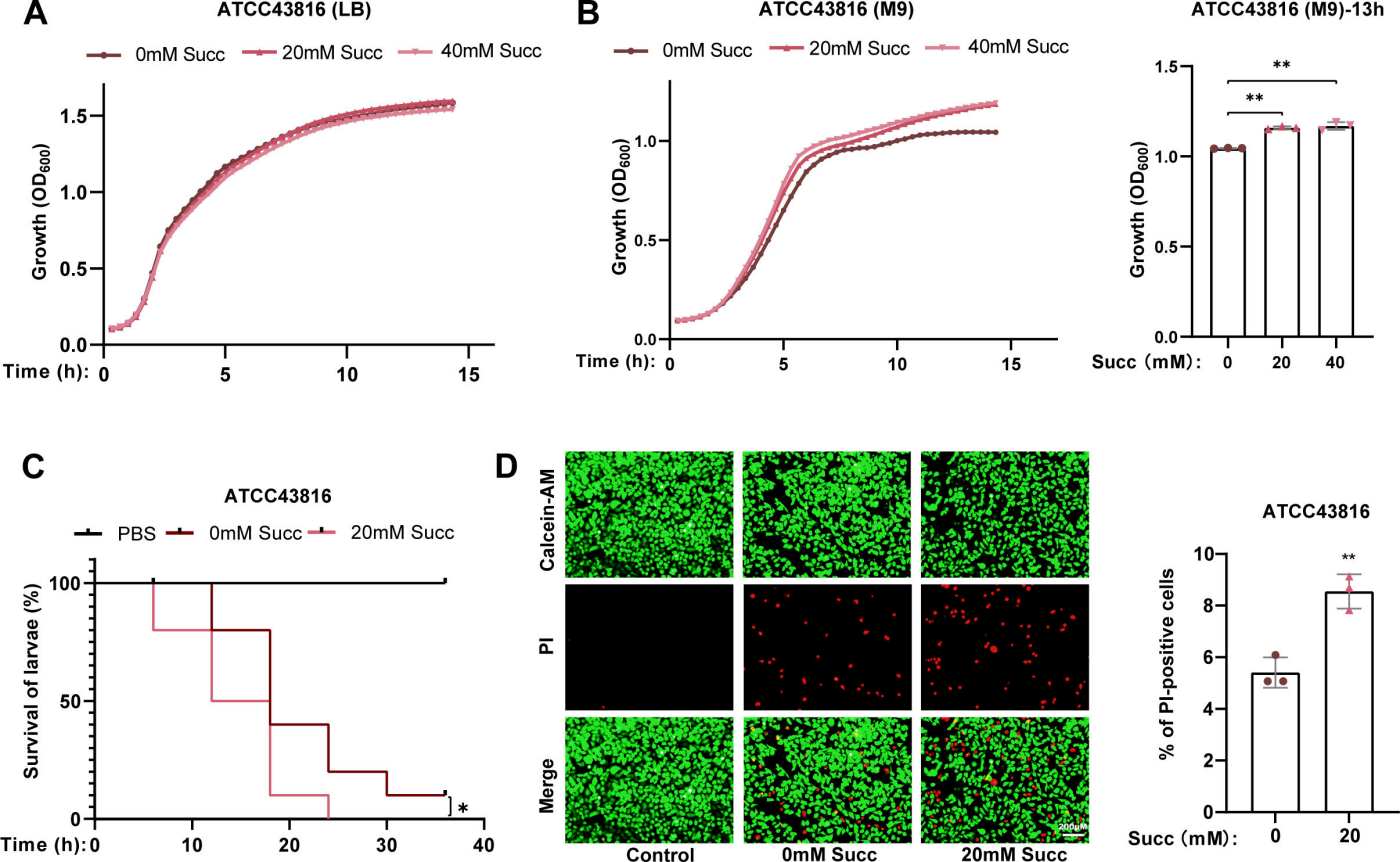
Succinate enhances the virulence and pathogenicity of hvKP. Growth curve of ATCC43816 in LB (**A**) or M9 medium (**B**) supplemented with different concentrations of succinate. (**C**) Toxicity assay of *Galleria mellonella* larvae infected with ATCC43816 pretreated with different concentrations of succinate. Each larva was injected with equal amounts of ATCC43816 pretreated with different concentrations of succinate. (**D**) Cytotoxicity assay of NCM460 cells infected by ATCC43816 pretreated with different concentrations of succinate. A living cell was stained with calcein-AM (green), and the dead cell was stained with propidium iodide (red). Original magnification, ×10. Scale bar, 200 µm. The cytotoxicity assay results were quantified based on the mortality percentage of NCM460 cells after being infected by ATCC43816 (multiplicity of infection = 130) for 12 h. PBS, phosphate-buffered saline; PI, propidium iodide; Succ, succinate. All data are presented as mean ± SD of three independent biological replicates. *P < 0.05, **P < 0.01.

As previous studies indicated that succinate facilitates pathogenicity of some clinical pathogenic bacteria, we first aimed to determine whether succinate has an effect on growth and virulence of hvKP. Bacteria used in this study were ATCC43816 (K2) and NTUH-K2044 (K1). In nutrient-rich medium (Luria-Bertani [LB]), succinate had no effect on the growth of ATCC43816 (Fig. 1A) and NTUH-K2044 (Fig. S1A). However, under conditions of nutrient-deficient medium (M9), the growth of ATCC43816 (Fig. 1B) or NTUH-K2044 (Fig. S1B) significantly increased by adding 20 mM succinate and further increased in the presence of 40 mM succinate, indicating that succinate may be an alternative nutrient to hvKP. In addition, the *Galleria mellonella* larvae toxicity assay demonstrated that pretreatment with succinate significantly enhanced the lethality of both ATCC43816 (Fig. 1C) and NTUH-K2044 (Fig. S1C). Consistent with these findings, succinate pretreatment also increased the mortality rates of NCM460 cells infected with ATCC43816 (Fig. 1D) and IEC6 cells infected with NTUH-K2044 (Fig. S1D). These results collectively indicate that succinate potentiates the virulence and pathogenicity of hvKP strains.

### Succinate promotes hvKP T6SS expression via DcuSR TCS

**Fig 2:**
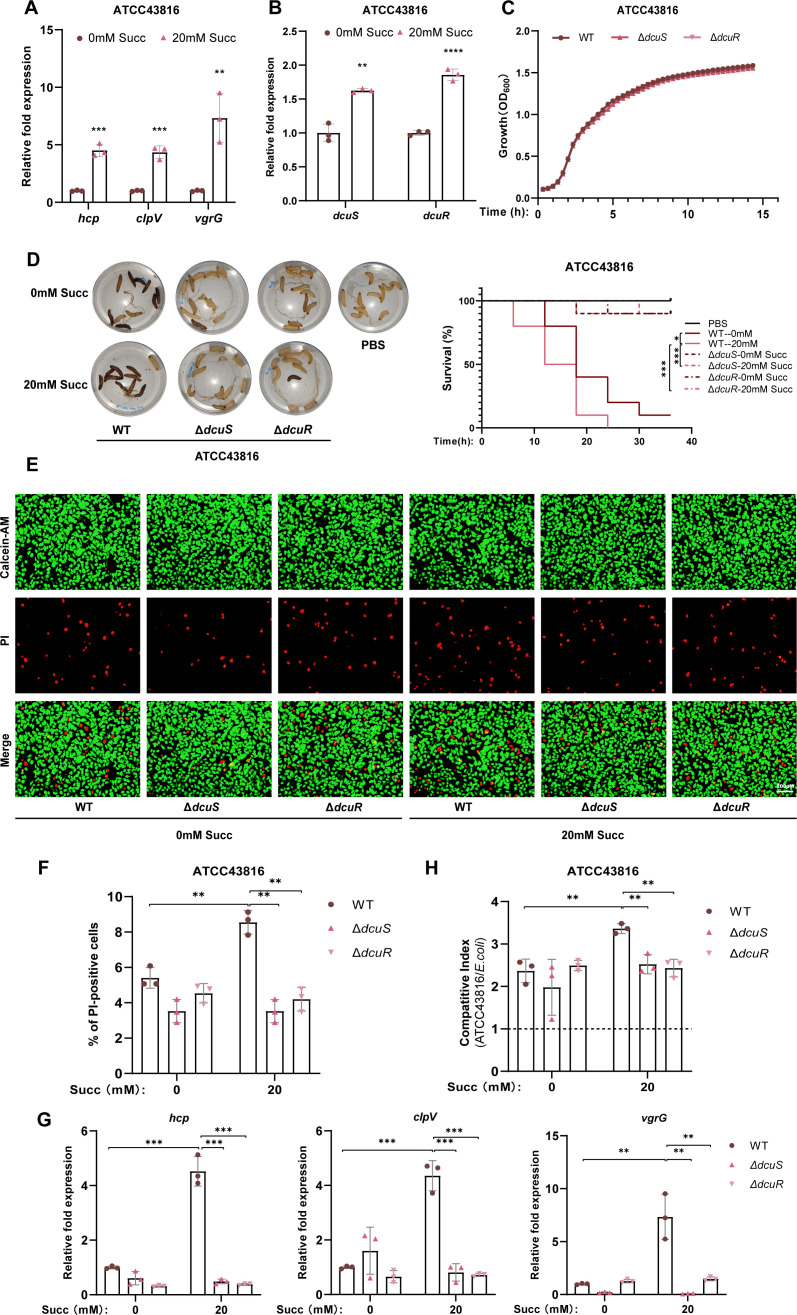
Succinate promotes hvKP T6SS expression via DcuSR TCS. (**A**) RT-qPCR was performed to measure the mRNA expression of *hcp*, *clpV*, and *vgrG* of ATCC43816 grown in LB medium supplemented with 0 or 20 mM succinate. (**B**) RT-qPCR was performed to measure the mRNA expression of *dcuS* and *dcuR* of ATCC43816 grown in LB medium supplemented with 0 or 20 mM succinate. (**C**) Growth curve of ATCC43816 WT, Δ*dcuS*, and Δ*dcuR* at 37°C in LB medium. (**D**) Toxicity assay of *Galleria mellonella* larvae infected with ATCC43816 WT, Δ*dcuS*, and Δ*dcuR* pretreated with 0 or 20 mM succinate. (**E**) Cytotoxicity assay of NCM460 cells infected with ATCC43816 WT, Δ*dcuS*, and Δ*dcuR* (multiplicity of infection = 130, 12 h) pretreated with 0 or 20 mM succinate. The cytotoxicity assay results were quantified based on the PI-positive percentage of NCM460 cells (**F**). (**G**) RT-qPCR to determine T6SS gene expression changes of ATCC43816 WT, Δ*dcuS*, and Δ*dcuR* in LB broth. (**H**) Competitive index analysis between ATCC43816 WT, Δ*dcuS*, Δ*dcuR*, and *E. coli* (MG1655) at a 1:1 ratio in LB medium with 0 or 20 mM succinate for 24 h. Δ*dcuR*, *dcuR* knockout mutant of ATCC43816; Δ*dcuS*, *dcuS* knockout mutant of ATCC43816; ATCC43816 WT, ATCC43816 wild-type strain; RT-qPCR, real-time quantitative polymerase chain reaction; Succ, succinate; WT, wild type. All data are presented as mean ± SD of three independent biological replicates. *P < 0.05, **P < 0.01, ***P < 0.001, ****P < 0.0001.

T6SS is a key virulence factor widely conserved in gram-negative bacteria, playing a pivotal role in host invasion (39). hvKP isolates associated with invasive infections have been reported to exhibit a high prevalence of T6SS genes (40–43). To investigate whether succinate mediates the T6SS gene expression, we analyzed the transcriptional levels of key T6SS structural genes (*hcp*, *clpV*, and *vgrG*) in ATCC43816 (Fig. 2A) and NTUH-K2044 (Fig. S2A). The results revealed a significant upregulation of these genes in the presence of 20 mM succinate, indicating that succinate enhances T6SS expression in hvKP.

Bioinformatic analyses revealed significant structural conservation between hvKP (ATCC43816/NTUH-K2044) and *E. coli* DcuS, with 56.33% sequence identity, 90.15% biochemical similarity, and substantial structural overlap (as assessed by the template modeling score [TM] score = 0.56). Most critically, all key residues in the C_4_-dicarboxylate-binding pocket (R107, H110, and R147; *E. coli* numbering) were strictly conserved (44, 45), strongly supporting functional equivalence as a succinate sensor (Fig. S3A). To further elucidate the regulatory role of the DcuSR TCS in this process, we examined the transcriptional response of *dcuS* and *dcuR* in ATCC43816. Both genes were significantly upregulated under 20 mM succinate conditions (Fig. 2B). We subsequently constructed *dcuS* and *dcuR* deletion mutants in ATCC43816, which exhibited growth rates comparable to the wild-type strain in both LB (Fig. 2C) and M9 media (Fig. S4A). Functional assays demonstrated that the deletion of *dcuS* or *dcuR* significantly attenuated the virulence of ATCC43816, as evidenced by improved survival rates of *Galleria mellonella* larvae (Fig. 2D) and NCM460 cells (Fig. 2E and F) following infection. Transcriptional analysis further confirmed that the loss of *dcuS* or *dcuR* abolished the succinate-mediated upregulation of T6SS genes, with no significant differences observed in T6SS gene expression between succinate-treated and untreated mutant strains (Fig. 2G).

Competitive assays revealed that both ATCC43816 and NTUH-K2044 exhibited significantly enhanced competitive fitness against *E. coli* in the presence of 20 mM succinate (Fig. 2H; Fig. S2B). However, this competitive advantage was completely abolished in *dcuS* or *dcuR* deletion mutants (Fig. 2H), indicating that the DcuSR TCS is essential for succinate-mediated competitive fitness. Collectively, these findings demonstrate that the DcuSR two-component system is indispensable for regulating both T6SS gene expression and the pathogenic potential of hvKP in response to succinate.

### T6SS is required for DcuSR-mediated virulence enhancement in hvKP

**Fig 3:**
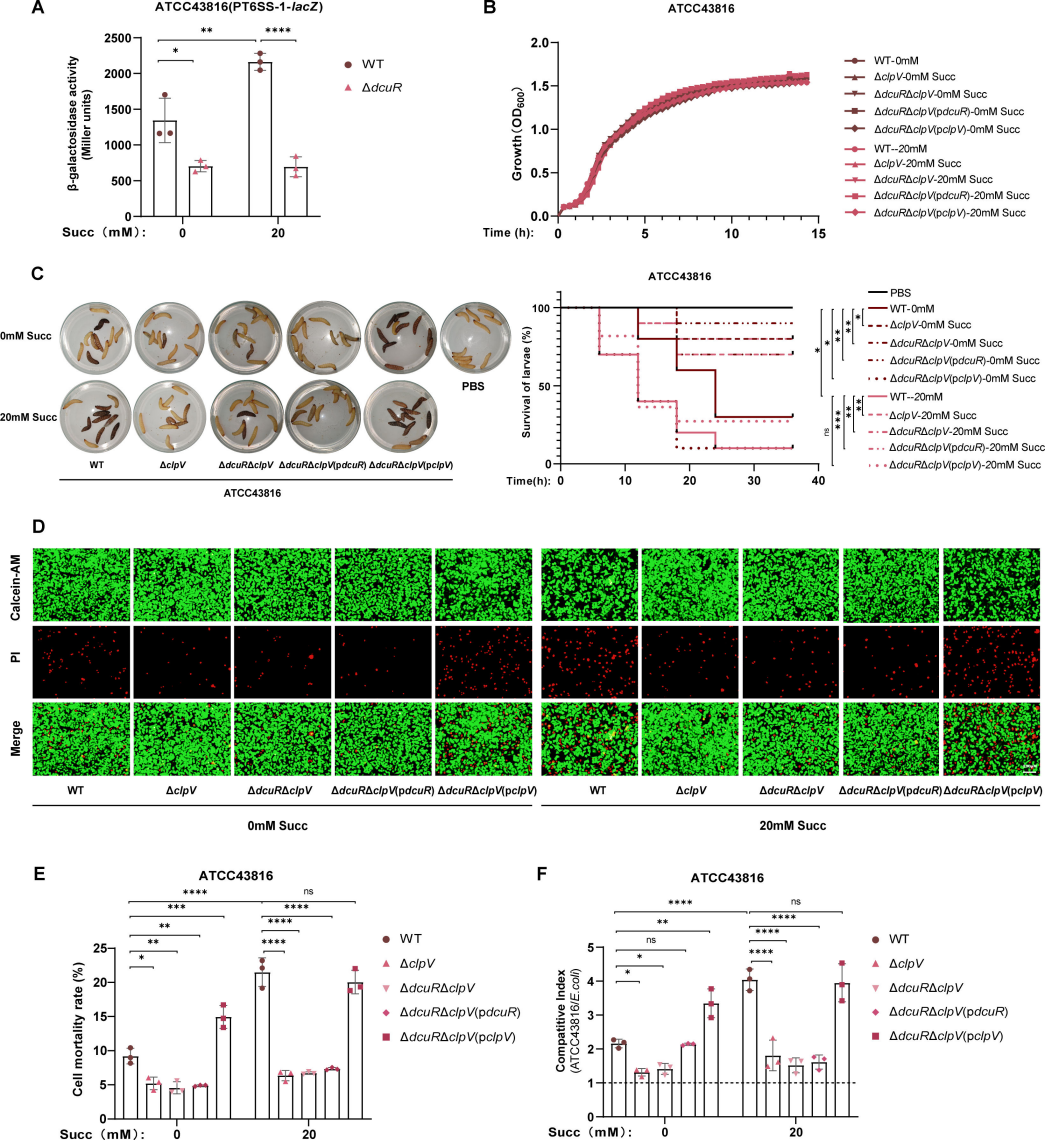
T6SS is required for DcuSR-mediated virulence enhancement in hvKP. (**A**) β-Galactosidase activity assay quantifying DcuR-dependent activation of the T6SS-1 promoter. (**B**) Growth curves of ATCC43816 WT, Δ*clpV*, Δ*dcuR*Δ*clpV*, Δ*dcuR*Δ*clpV*(p*dcuR*), and Δ*dcuR*Δ*clpV*(p*clpV*) at 37°C in LB medium with 0 or 20 mM succinate. (**C**) Toxicity assay of *Galleria mellonella* larvae infected with ATCC43816 WT, Δ*clpV*, Δ*dcuR*Δ*clpV*, Δ*dcuR*Δ*clpV*(p*dcuR*), and Δ*dcuR*Δ*clpV*(p*clpV*) pretreated with 0 or 20 mM succinate. (**D**) Cytotoxicity assay of NCM460 cells infected with ATCC43816 WT, Δ*clpV*, Δ*dcuR*Δ*clpV*, Δ*dcuR*Δ*clpV*(p*dcuR*), and Δ*dcuR*Δ*clpV*(p*clpV*) (multiplicity of infection = 130, 12 h). Cytotoxicity was quantified by measuring the percentage of PI-positive NCM460 cells (**E**). (**F**) Competitive index analysis of ATCC43816 strains [WT, Δ*clpV*, Δ*dcuR*Δ*clpV*, Δ*dcuR*Δ*clpV*(p*dcuR*), and Δ*dcuR*Δ*clpV*(p*clpV*)] against *E. coli* (MG1655) at a 1:1 ratio in LB medium supplemented with 0 or 20 mM succinate for 24 h. Δ*clpV*, *clpV* knockout mutant of ATCC43816; Δ*dcuR*Δ*clpV*, *dcuR*/*clpV* double mutant of ATCC43816; Δ*dcuR*Δ*clpV*(p*dcuR*), *dcuR*-complemented strain of *dcuR*/*clpV* double mutant; Δ*dcuR*Δ*clpV*(p*clpV*), *clpV*-complemented strain of *dcuR*/*clpV* double mutant; ATCC43816 WT, ATCC43816 wild-type strain; Succ, succinate. All data are presented as mean ± SD of three independent biological replicates. *P < 0.05, **P < 0.01, ***P < 0.001, ****P < 0.0001.

Initial RT-qPCR analyses demonstrated that deletion of *dcuS* or *dcuR* abolished the succinate-mediated upregulation of T6SS genes. To determine whether DcuR can activate the T6SS promoter, we constructed a reporter system by cloning the T6SS-1 promoter upstream of the β-galactosidase reporter gene *lacZ* in the pME6522 reporter plasmid. β-Galactosidase activity assays revealed that the deletion of *dcuR* suppressed T6SS-1 promoter activity regardless of succinate stimulation, confirming DcuR-dependent transcriptional regulation of T6SS (Fig. 3A).

In addition, to definitively establish T6SS-dependence in DcuSR-mediated virulence enhancement in hvKP, we constructed T6SS knockout and complementation mutants for functional characterization, which exhibited growth rates comparable to the wild-type strain in LB medium supplemented with 0 or 20 mM succinate (Fig. 3B). Functional analyses revealed that T6SS inactivation (Δ*clpV*) significantly attenuated ATCC43816 virulence with or without succinate pretreatment, as demonstrated by improved survival of *Galleria mellonella* larvae (Fig. 3C) and reduced cytotoxicity in NCM460 cells (Fig. 3D and E). Furthermore, virulence attenuation in the Δ*dcuR*Δ*clpV* double mutant mirrored that in the Δ*clpV* single mutant, showing comparable outcomes in both *Galleria mellonella* larvae assays and NCM460 cytotoxicity tests. Genetic complementation assays revealed that *clpV* expression in the Δ*dcuR*Δ*clpV* double mutant fully restored virulence phenotypes, including *Galleria mellonella* larval killing efficiency and NCM460 cell damage. In contrast, *dcuR* complementation failed to reverse the virulence attenuation observed in the Δ*dcuR*Δ*clpV* double mutant (Fig. 3C through E).

Similarly, competitive assays demonstrated that both Δ*clpV* and Δ*dcuR*Δ*clpV* exhibited significantly attenuated competitive fitness against *E. coli* compared to the wild type (WT) in the presence of 0 or 20 mM succinate (Fig. 3F). Moreover, complementation of the Δ*dcuR*Δ*clpV* double mutant with *clpV* extremely enhanced the competitive fitness against *E. coli* in the presence of 0 or 20 mM succinate, whereas no apparent changes were observed when the Δ*dcuR*Δ*clpV* double mutant was complemented with *dcuR* (Fig. 3F). Collectively, T6SS is required for DcuSR-mediated virulence enhancement in hvKP.

### Succinate enhances hvKP adherence to intestinal epithelial cells by upregulating type III fimbrial gene expression via DcuSR TCS

T6SS is known to target and attack host cells through a contact-dependent mechanism, while fimbriae adhesins are well-established virulence factors that facilitate bacterial adherence and are closely associated with host colonization and pathogenicity (46, 47). As our previous results indicated that hvKP utilizes succinate to enhance T6SS expression, we further investigated whether succinate also promotes hvKP adherence to epithelial cells by modulating fimbria expression.

**Fig 4:**
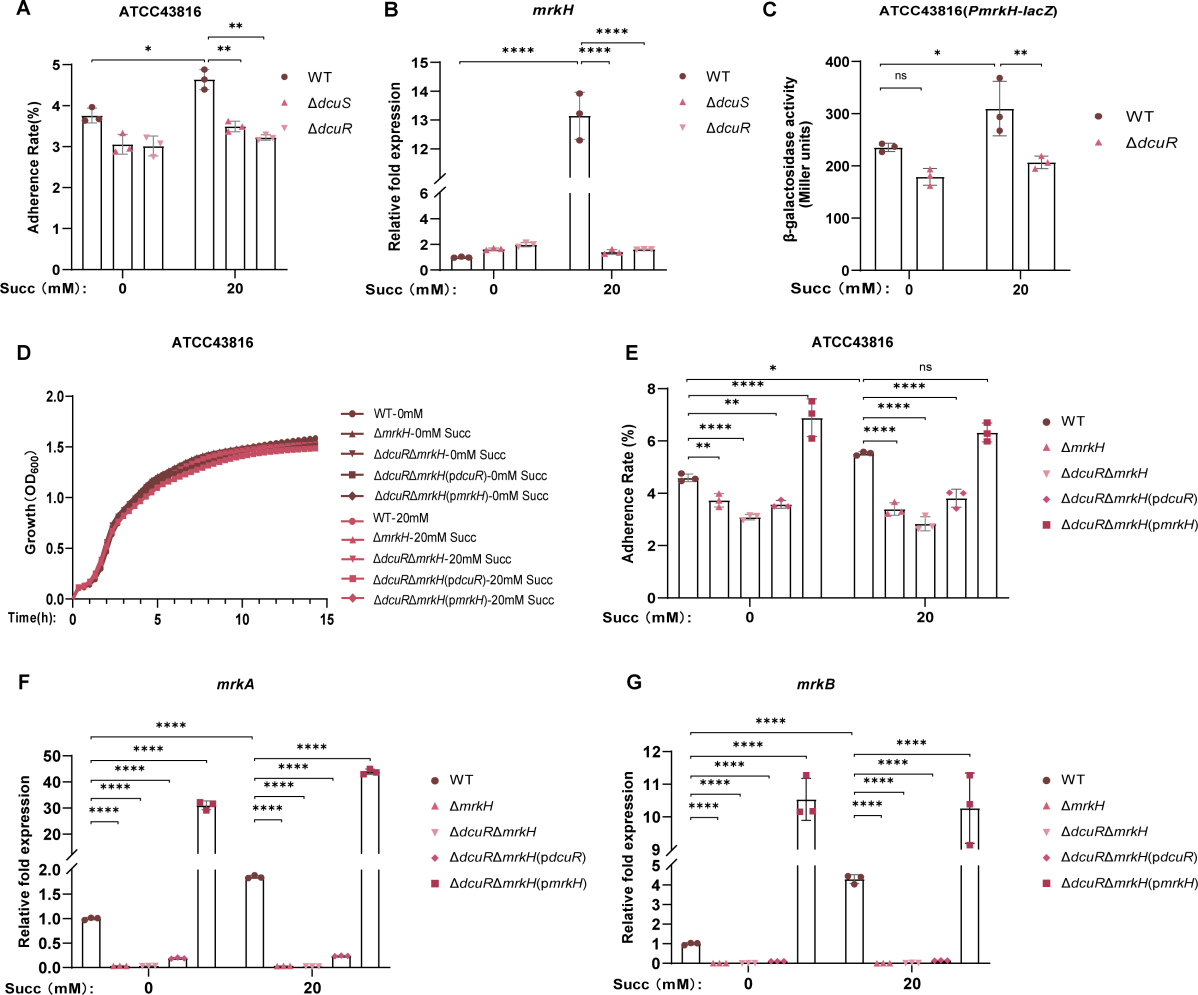
Succinate enhances hvKP adherence to intestinal epithelial cells by upregulating type III fimbrial gene expression via DcuSR TCS. (**A**) Adherence rate of ATCC43816 WT, Δ*dcuS*, and Δ*dcuR* to NCM460 cells. (**B**) Fold expression changes in the *mrkH* gene of ATCC43816 WT, Δ*dcuS*, and Δ*dcuR* in LB broth treated with or without 20 mM succinate. (**C**) β-Galactosidase activity assay quantifying DcuR-dependent activation of the *mrkH* promoter. (**D**) Growth curves of ATCC43816 WT, Δ*mrkH*, Δ*dcuR*Δ*mrkH*, Δ*dcuR*Δ*mrkH*(p*dcuR*), and Δ*dcuR*Δ*mrkH*(p*mrkH*) at 37°C in LB medium with 0 or 20 mM succinate. (**E**) Adherence rate of ATCC43816 WT, Δ*mrkH*, Δ*dcuR*Δ*mrkH*, Δ*dcuR*Δ*mrkH*(p*dcuR*), Δ*dcuR*Δ*mrkH*(p*mrkH*) to NCM460 cells. (**F and G**) Relative fold expression of *mrkA* and *mrkB* gene of ATCC43816 WT, Δ*mrkH*, Δ*dcuR*Δ*mrkH*, Δ*dcuR*Δ*mrkH*(p*dcuR*), and Δ*dcuR*Δ*mrkH*(p*mrkH*) in LB medium treated with 0 or 20 mM succinate. Δ*dcuR*, *dcuR* knockout mutant of ATCC43816; Δ*dcuR*Δ*mrkH*, *dcuR*/*mrkH* double mutant of ATCC43816; Δ*dcuR*Δ*mrkH*(p*dcuR*), *dcuR*-complemented strain of *dcuR*/*mrkH* double mutant; Δ*dcuR*Δ*mrkH*(p*mrkH*), *mrkH*-complemented strain of *dcuR*/*mrkH* double mutant; Δ*dcuS*, *dcuS* knockout mutant of ATCC43816; Δ*mrkH*, *mrkH* knockout mutant of ATCC43816; ATCC43816 WT, ATCC43816 wild-type strain; Succ, succinate. All data are presented as mean ± SD of three independent biological replicates. *P < 0.05, **P < 0.01, ****P < 0.0001.

Adhesion assays revealed that pretreatment with 20 mM succinate significantly enhanced the adherence capacity of both ATCC43816 (Fig. 4A) and NTUH-K2044 (Fig. S5A) to NCM460 intestinal epithelial cells. Transcriptional analysis demonstrated that succinate markedly upregulated the expression of key fimbrial genes, including *fimH* (encoding type I fimbriae) (Fig. S6A) and *mrkH* (regulating type III fimbriae) (Fig. 4B), in both ATCC43816 and NTUH-K2044 (Fig. S5B) compared to untreated controls. The adhesion advantage conferred by succinate in the ATCC43816 wild-type strain was completely abolished in *dcuS* and *dcuR* deletion mutants (Fig. 4A). Consistent with this observation, both Δ*dcuS* and Δ*dcuR* mutants exhibited significantly downregulated expression of *fimH* (Fig. S6A) and *mrkH* (Fig. 4B), regardless of succinate treatment. To elucidate the contributions of type I and type III fimbriae in mediating succinate-enhanced adhesion, Δ*fimH* and Δ*mrkH* mutants in ATCC43816 were constructed. The Δ*fimH* (Fig. S6B) and Δ*mrkH* (Fig. 4D) mutants exhibited growth curves in LB medium comparable to the WT regardless of succinate. Remarkably, *fimH* deletion had no significant effect on adhesion capacity (Fig. S6C), while *mrkH* knockout resulted in adhesion defects that were not rescued by succinate treatment (Fig. 4E). These results indicate that succinate enhances hvKP adhesion primarily through type III fimbriae, with minimal contribution from type I fimbriae.

Furthermore, β-galactosidase activity assays revealed that the *mrkH* promoter activity was significantly enhanced by 20 mM succinate, whereas this enhancement was markedly attenuated by *dcuR* deletion, establishing DcuR as a transcriptional activator of *mrkH* (Fig. 4C). To further delineate the DcuR-MrkH functional axis in type III fimbriae-mediated adhesion, we generated the Δ*dcuR*Δ*mrkH* double mutant, along with its complemented strains in ATCC43816, all of which exhibited WT-comparable growth kinetics in LB medium with or without succinate (Fig. 4D). The Δ*dcuR*Δ*mrkH* double mutant recapitulated the adhesion defect observed in the Δ*mrkH* mutant, displaying impaired adhesion to NCM460 cells regardless of succinate pretreatment (Fig. 4E). RT-qPCR analysis revealed coordinated downregulation of *mrkA* and *mrkB* expression in both Δ*mrkH* and Δ*dcuR*Δ*mrkH* mutants (Fig. 4F and G). Notably, genetic complementation demonstrated that *mrkH* (not *dcuR*) significantly rescued adherence capacity and *mrkA*/*B* expression under 20 mM succinate induction. Collectively, these results establish type III fimbriae as the principal mediator of succinate-enhanced intestinal epithelial cell adherence in hvKP via DcuSR.

### hvKP utilizes succinate as an important substrate to promote ATP production

**Fig 5:**
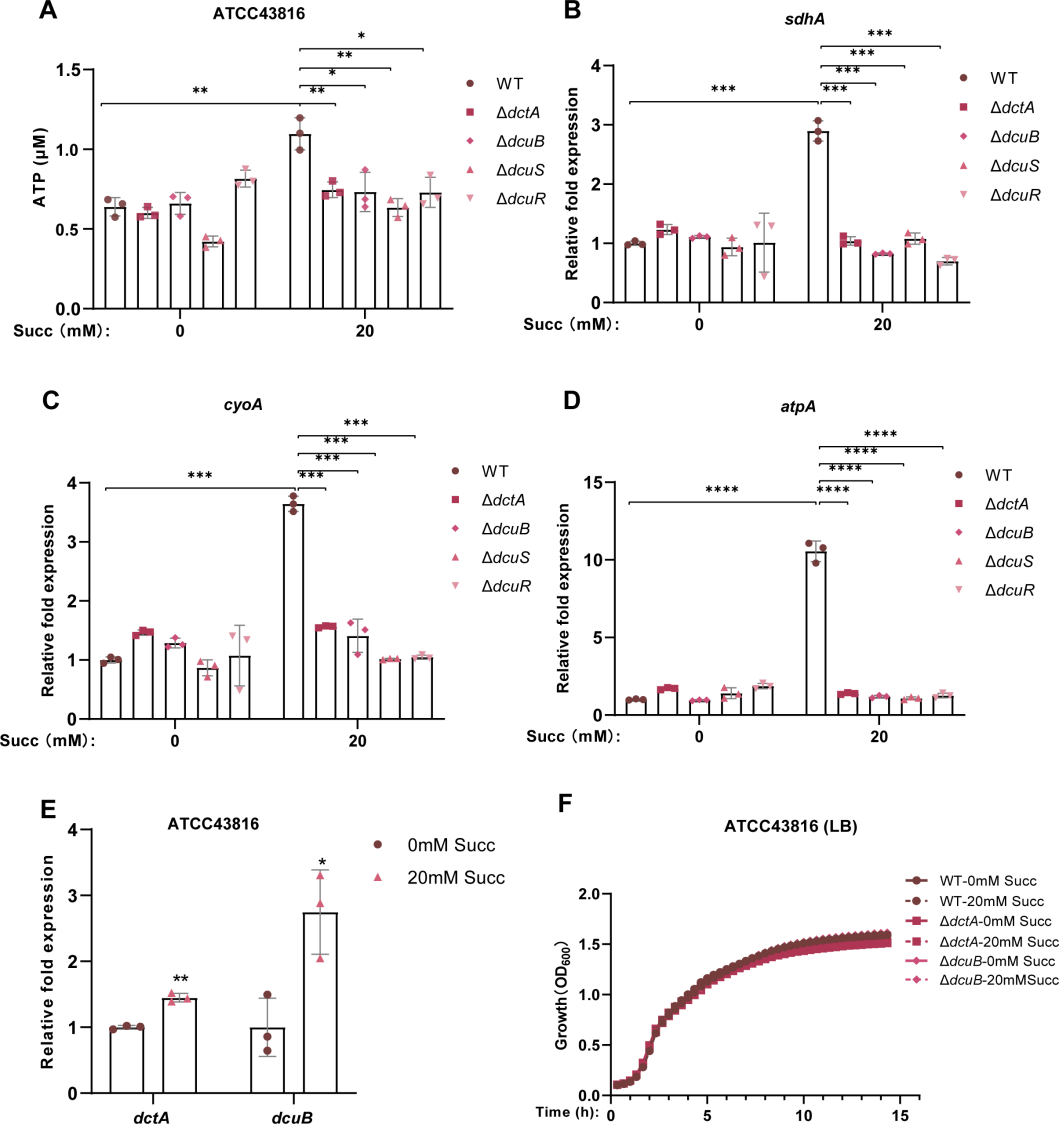
hvKP utilizes succinate as an important substrate to promote ATP production. (**A**) ATP production of ATCC43816 WT, Δ*dctA*, Δ*dcuB*, Δ*dcuS*, and Δ*dcuR* in LB medium supplemented with 0 or 20 mM succinate. (**B–D**) Fold expression changes in *sdhA*, *atpA*, and *cyoA* genes of ATCC43816 WT, Δ*dctA*, Δ*dcuB*, Δ*dcuS*, and Δ*dcuR* in LB medium treated with or without 20 mM succinate. (**E**) RT-qPCR to determine dctA, dcuB mRNA expression level of ATCC43816 in LB medium supplemented with 0 or 20 mM succinate. (**F**) Growth curve of ATCC43816 WT, Δ*dctA*, and Δ*dcuB* in LB medium supplemented with 0 or 20 mM succinate. Succ, succinate; ATCC43816 WT, ATCC43816 wild-type strain; Δ*dctA*, *dctA* knockout mutant of ATCC43816; Δ*dcuB*, *dcuB* knockout mutant of ATCC43816; Δ*dcuR*, *dcuR* knockout mutant of ATCC43816; Δ*dcuS*, *dcuS* knockout mutant of ATCC43816. All data are presented as mean ± SD of three independent biological replicates. *P < 0.05, **P < 0.01, ***P < 0.001, ****P < 0.0001.

Succinate, as an intermediate product of the TCA cycle, has a well-established role in cellular metabolism. However, whether succinate exerts an influence on the TCA cycle of hvKP remains elusive. Therefore, in this section, we primarily set out to explore the potential impact of succinate on the TCA cycle, electron transport chain (ETC), and ATP production of hvKP. Our results demonstrated that the ATP production of ATCC43816 was significantly enhanced in the presence of 20 mM succinate (Fig. 5A). Furthermore, the expression of *sdhA* (encoding succinate dehydrogenase flavoprotein subunit in TCA cycle), *cyoA* (encoding cytochrome o ubiquinol oxidase subunit II in ETC), and *atpA* (encoding ATP synthase subunit alpha in ETC), all of which are associated with ATP synthesis, was significantly upregulated by the presence of 20 mM succinate in both ATCC43816 (Fig. 5B through D) and NTUH-K2044 (Fig. S8A).

Additionally, the expression of succinate transporters *dctA* and *dcuB* was upregulated in the presence of 20 mM succinate (Fig. 5E; Fig. S8B). Structural comparisons with *E. coli* revealed that DctA/DcuB in hvKP maintain >93% sequence identity and near-complete structural conservation (TM score > 0.98), suggesting potential functional conservation in succinate transport (Fig. S3). To further explore the roles of these transporters in hvKP’s utilization of succinate and their impact on the TCA cycle, ETC, and ATP production, we constructed *dctA* or *dcuB* deletion mutations in ATCC43816. No significant differences in growth curves were observed between the mutant strains and the wild-type ATCC43816 in LB (Fig. 5F) or M9 (Fig. S7A and B) broth, regardless of whether 20 mM succinate was present or not. However, the upregulation of ATP production and the expression of *sdhA*, *atpA*, and *cyoA* induced by 20 mM succinate in the wild-type ATCC43816 were completely abolished by the deletion of C_4_-DC transporter gene *dctA* or *dcuB* (Fig. 5A through D). These results suggest that succinate promotes hvKP ATP production, and this boosting effect may be achieved by enhancing the TCA cycle and ETC activity.

Given the important role of DcuSR TCS in the regulation of C_4_-DC metabolism (48–51), we next investigated whether it regulates hvKP’s utilization of succinate to enhance the TCA cycle. The Δ*dcuS* and Δ*dcuR* mutants of ATCC43816 exhibited similar inhibitory effects on ATP production and the expression of ATP synthesis-related genes, even after treatment with 20 mM succinate (Fig. 5A through D). However, whether the DcuSR TCS promotes hvKP ATP production by regulating the expression of succinate transporters DctA and DcuB remains to be further verified.

## DISCUSSION

Intestinal colonization by hvKP is a critical determinant of clinical pathogenicity, enabling systemic dissemination and invasive infections (52, 53). The gut microenvironment is enriched with diverse metabolites from both host and microbiota (54–56). hvKP must develop strategies to utilize the metabolites to overcome colonization resistance caused by gut commensal microbiota and enhance pathogenicity toward intestinal epithelial cells (57, 58). In this study, we focus on the gut metabolite succinate, which can accumulate in the gut under various medical conditions, such as diabetes (59, 60) and inflammatory bowel disease (61), and may facilitate hvKP colonization and virulence. Notably, diabetic patients exhibit increased susceptibility to hvKP infections and higher incidence of *Klebsiella pneumoniae* liver abscesses (62–64), suggesting a potential link between host metabolic dysregulation and hvKP virulence. Furthermore, succinate has been reported to promote virulence expression in enteric pathogens like *Salmonella* Typhimurium and enterohemorrhagic *E. coli* (30, 31). Based on these findings, we hypothesized that succinate may similarly enhance hvKP pathogenicity by coordinating virulence expression and metabolic adaptation.

**Fig 6:**
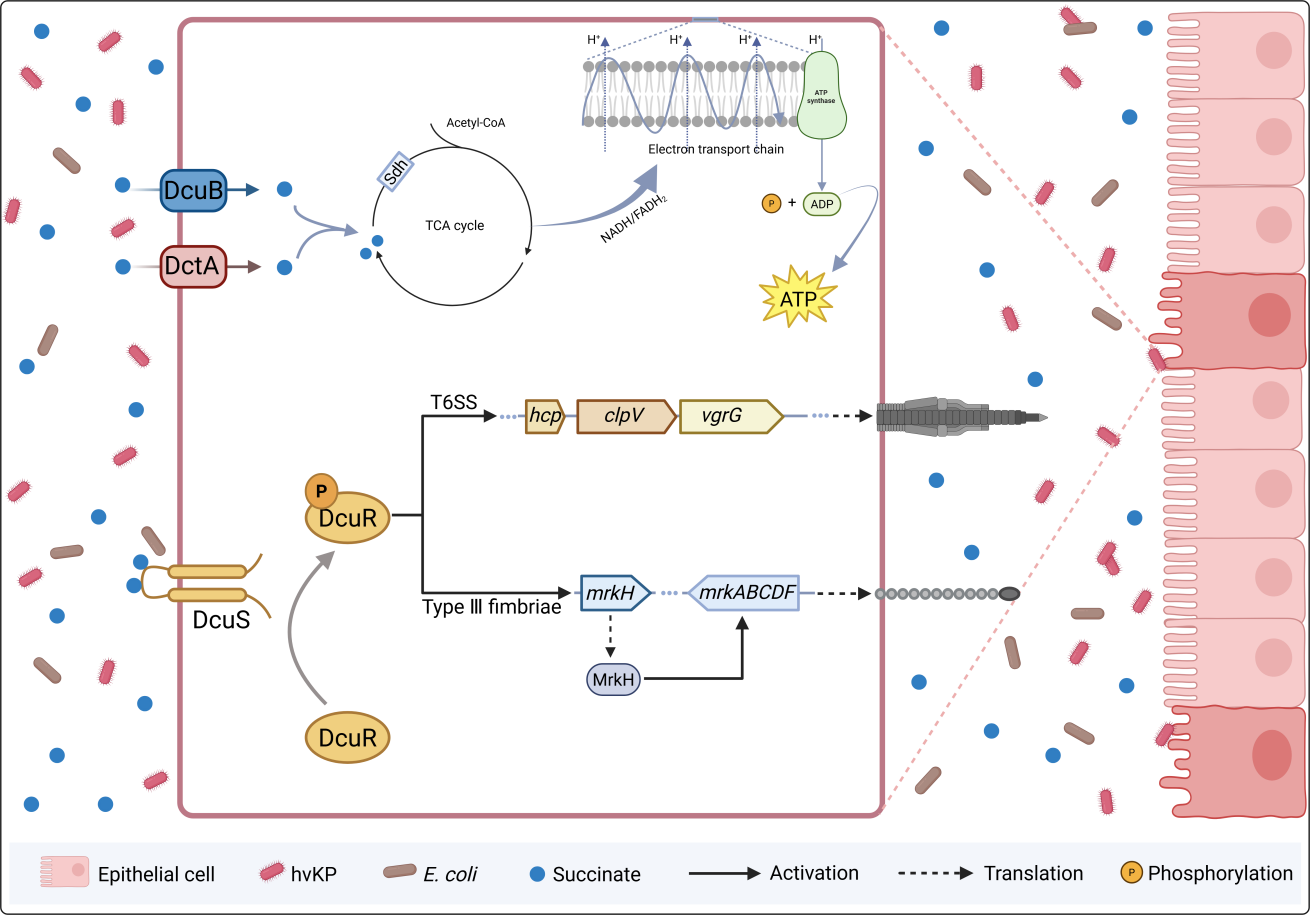
Proposed model for succinate-mediated enhancement of hvKP pathogenicity. The schematic illustration was created using BioRender (BioRender.com). As a signaling molecule, succinate may be detected by the TCS sensor kinase DcuS, triggering its autophosphorylation and subsequent transfer of phosphate groups to the response regulator DcuR. Phosphorylated DcuR could activate the expression of hvKP T6SS genes, which might increase the cytotoxicity of hvKP toward intestinal epithelial cells and may enhance competitive advantage against commensal *E. coli*. Additionally, phosphorylated DcuR might upregulate *mrkH* (regulating type III fimbriae), which may promote the adhesion of hvKP to intestinal epithelial cells. As a metabolic substrate, succinate could be imported via the transporters DctA and DcuB. Once internalized, succinate may be catabolized to drive ATP synthesis, potentially supporting bacterial energy demands, and this boosting effect may be achieved by enhancing the TCA cycle and ETC activity. This model integrates genetic and phenotypic observations with hypothesized mechanisms that require further experimental validation.

Here, we establish that the succinate-DcuSR axis in hvKP coordinately activates virulence factor production and supports bioenergetic adaptation. A proposed model for this succinate-mediated pathogenesis effects in hvKP is presented in Fig. 6. Mechanistically, extracellular succinate may be sensed by the DcuSR TCS, possibly triggering phosphorylation of DcuR and subsequent upregulation of type III fimbriae and T6SS. These virulence factors collectively enhance hvKP adhesion and cytotoxicity to intestinal epithelial cells and competitive fitness against commensal *E. coli*. Functionally, succinate might be imported via the transporters DctA and DcuB to promote hvKP ATP production, potentially through augmentation of the TCA cycle and increased ETC activity.

The DcuSR TCS, known for its role in C_4_-dicarboxylate sensing and regulation, has primarily been studied in *E. coli* (27, 34–38). Notably, Liu et al. demonstrated that DcuSR in EHEC activates the T3SS via L-malate sensing to enhance virulence (37). Although hvKP lacks T3SS, our study reveals a conserved regulatory logic: succinate-activated DcuSR in hvKP upregulates the T6SS, a functional analog of T3SS that mediates host cytotoxicity and interbacterial competition through effector delivery (65). This evolutionary convergence underscores the versatility of DcuSR in coupling metabolite sensing to virulence expression across gram-negative pathogens. Our findings indicate that succinate not only promotes the pathogenicity by activating DcuSR TCS but also strengthens the competitiveness against *E. coli*, aligning with the critical role of T6SS in hvKP gut colonization through interbacterial competition and its indirect contribution to pathogenicity (66). Beyond T3SS and T6SS, whether succinate and other C_4_-dicarboxylates modulate other secretory systems (types I–IX) (67, 68) through the DcuSR TCS activation warrants further investigation.

Besides T6SS, this study reveals that succinate also promotes the hvKP adhesion capacity to intestinal epithelial cells. Our experimental results further demonstrate that type III fimbriae, but not type I fimbriae, mediate succinate-enhanced adhesion of ATCC43816 to intestinal epithelial cells. This finding aligns with previous reports showing type I fimbriae are dispensable for gut colonization in ATCC43816 (69), and type III fimbriae play a dominant role in adhesion compared to type I fimbriae (70). However, given that there are still different opinions on the roles of type I and type III fimbriae in different hvKPs, further exploration is needed in the future (71). Moreover, fimbriae have been reported to affect the bacterium’s secretion system. Otto et al. (72) explored the role of type IV pili in facilitating T6SS-mediated lethal efficiency in *Vibrio cholerae* by promoting autoaggregation. Given the concurrent upregulation of T6SS and type III fimbriae-mediated adhesion, we hypothesize that type III fimbriae-mediated adhesion potentiates T6SS effector delivery, thereby amplifying cytotoxicity against intestinal epithelium and competitive suppression of commensals. However, further evidence is required to confirm this hypothesis.

Our findings reveal that succinate selectively promotes hvKP proliferation in nutrient-limited M9 medium but not nutrient-rich LB broth, indicating its role as a critical metabolic substrate under starvation conditions, a functional trait shared with another C_4_-DC (e.g., fumarate, L-malate, and L-aspartate) in Enterobacteriaceae (35–38). While deletion of the succinate transporter *dctA* or *dcuB* did not impair hvKP growth in succinate-supplemented M9 medium, this likely reflects functional redundancy between DctA and DcuB, or the promoting effect of succinate on hvKP in nutrient-deficient medium may be affected by many complicated aspects. Intriguingly, in LB medium—where succinate does not enhance growth—the absence of *dctA*, *dcuB*, *dcuS*, or *dcuR* abolished succinate-driven ATP synthesis in wild-type hvKP. This metabolic impairment aligns with the observed downregulation of T6SS and type III fimbriae expression, suggesting a convergent mechanism linking energy metabolism to virulence activation. Moreover, given that ATP hydrolysis powers T6SS effector translocation (73) and fimbrial assembly (74, 75), we assume that succinate-induced ATP synthesis synergizes with DcuSR-mediated virulence gene expression to amplify hvKP cytotoxicity. Future studies using ATP synthase inhibitors could delineate the causal relationship between energy metabolism and virulence effector function.

In addition to its role in metabolism, succinate functions as a signaling molecule by binding to the succinate receptor (SUCNR1) on the cell membrane, thereby modulating various host cell regulatory processes, such as promoting inflammatory response (76) and participating in post-translational modification (77). To elucidate the impact of succinate on the virulence of hvKP, we pretreated hvKP with succinate before infecting host cells at a constant multiplicity of infection (MOI) in our cytotoxicity assays. However, this study has certain limitations. First, while sequence analyses indicate conserved functions, the succinate-sensing capability of DcuS and transport activities of DctA/DcuB in hvKP require direct experimental validation through approaches such as ligand-binding assays (e.g., isothermal titration calorimetry or surface plasmon resonance for DcuS) and isotopic tracer studies (for DctA/DcuB). Second, although our β-galactosidase reporter assays demonstrated DcuR-dependent transcriptional activation of T6SS/*mrkH*, we acknowledge that additional direct binding evidence (e.g., electrophoretic mobility shift assay or chromatin immunoprecipitation quantitative PCR) would further strengthen our mechanistic conclusions. Third, our findings are primarily based on *in vitro* cell infection and *Galleria mellonella* larvae experiments, which, while providing mechanistic insight, may not fully capture the complexity of hvKP infection dynamics in mammalian hosts. Further studies should establish robust evidence in murine models to validate whether succinate enhances the capacity of hvKP to induce invasive and metastatic infections.

In conclusion, our findings provide critical insights into the evolutionary adaptation of hvKP in utilizing C_4_-DC succinate as both a metabolic substrate and a signaling molecule to regulate virulence expression and enhance pathogenicity. Genetic evidence demonstrates that the DcuSR system mediates succinate-dependent upregulation of T6SS and type III fimbriae, while functional DctA/DcuB systems are required for succinate-associated ATP enhancement. Given that the human gut is a complex ecosystem enriched with metabolites derived from both the microbiota and the host, further investigations are necessary to determine whether additional gut metabolites influence hvKP virulence and pathogenicity. Our findings highlight the potential of targeting the DcuSR TCS as a novel therapeutic strategy for preventing and controlling hvKP-associated invasive and metastatic infections.

## MATERIALS AND METHODS

### Bacterial strains, plasmids, and primers

The strains and plasmids used in this study are shown in Table S1. Knockout strains were generated using the λ Red recombinase system, following standard molecular cloning procedures (78). Plasmid pIJ773 was used as template to amplify the apramycin resistance gene and FRT sites at both ends with primers containing 60 bp of upstream or downstream homologous sequences of the target gene. Plasmid pACBSR-Hyg containing the λ Red recombinase system was used to promote homologous recombination of the apramycin resistance fragment and the target gene. Plasmid pFLP-Hyg containing FLP recombinase was used to excise the apramycin resistance fragment. Plasmid pACBSR-Hyg and pFLP-Hyg were resistant to hygromycin and were automatically lost when cultured at 43°C for 2–3 days. Overexpression strains were generated using the plasmid pSTVA (apramycin-resistant plasmid derived from pSTV28). The full-length coding sequence of target genes was PCR-amplified and cloned into the EcoRI restriction site of pSTVA. Recombinant plasmids were transformed into hvKP recipient strains via electroporation. Transcriptional reporter plasmids were generated by inserting PCR-amplified promoter regions into the EcoRI/BstI site of pME6522, a promoter-probe vector containing a promoter-less *lacZ* gene and tetracycline resistance. All strains were verified by PCR amplification and agarose gel electrophoresis. The PCR products with correct electrophoresis bands were further confirmed by first-generation sequencing. Primers used for mutant construction and verification are listed in Table S2. The bacterial strains were grown at 37°C in LB medium, M9 medium (1× M9 salts, 2 mM MgSO_4_, 0.1 mM CaCl_2_, and 0.4% glucose) or Dulbecco's modified Eagle medium. Antibiotics were added when necessary, with the final concentrations of apramycin 50 µg/mL, hygromycin 100 µg/mL, and tetracycline 50 µg/mL.

### Bacterial adherence experiments

Bacterial adherence experiments were performed with reference to previous methods with minor modifications (79). NCM460 and IEC6 cells were stored in our laboratory. NCM460 and IEC6 cells were cultured in 5% CO_2_ at 37°C to a suitable density and then passed through 24-well plates for 12 h. Before infection, NCM460 or IEC6 cells were washed three times with phosphate-buffered saline (PBS) and added with fresh 1640 or DMEM with 1% fetal bovine serum (FBS). Secondary enrichment to the logarithmic phase of bacteria strains (with or without succinate pretreatment) was then used to infect the cell monolayers at an MOI of 100:1. After incubation for 3 h in 5% CO_2_ at 37°C, culture medium containing unattached bacteria was removed. The cells were washed three times by PBS and then were lysed with 0.2% Triton. The lysate was diluted and applied to LB agar plates to calculate colony-forming units (CFUs). Adherence rate was calculated to reflect the adhesion ability of bacteria.

### *Galleria mellonella* larvae assays

*Galleria mellonella* larvae were used to evaluate the *in vivo* toxicity of bacteria. Logarithmic bacterial strains (with different concentrations of succinate pretreatment) were centrifuged and washed with PBS. Bacteria resuspended in PBS were injected into *Galleria mellonella* larvae at standardized inocula: 1 × 10^4^ CFU/larva for ATCC43816 and 1 × 10^3^ CFU/larva for NTUH-K2044. The survival of *Galleria mellonella* larvae was observed every 6 h. The survival curves were drawn.

### Cytotoxicity assays

Cytotoxicity was tested by cell calcein (CA)-AM/propidium iodide (PI) staining. NCM460 or IEC6 cells were grown in 12-well culture plates containing 1640/DMEM supplemented with 10% FBS and incubated at 37°C in 5% CO_2_ overnight to stick wall completely. The wells were washed with PBS three times, and fresh 1640/DMEM supplemented with 1% FBS was added. Bacterial strains were cultured in LB medium at 37°C overnight, and then 200 µL bacterial culture was added into fresh medium for secondary culture to the logarithmic growth stage with or without succinate pretreatment. After centrifugation and washing, the bacterial strains were resuspended with PBS. Bacterial strains infected the cells at an appropriate MOI for 12 h at 37°C in 5% CO_2_. NTUH-K2044 infected IEC6 cells with an MOI of 100; ATCC43816 infected NCM460 cells with an MOI of 130. Then, 2 µM CA-AM was used to visualize living cells, and 4 µM PI was used to stain nuclei of dead cells. After incubation at 37°C from light for 10–20 min, stained cells were observed by the inverted fluorescence microscope (Olympus IX71, Olympus Corporation) under 490 or 535 nm excitation laser.

### *In vitro* competition assays

*In vitro* competition assays were performed between hvKP (NTUH-K2044, ATCC43816 WT, Δ*dcuS*, and Δ*dcuR*) and MG1655. A plasmid containing a green fluorescent protein reporter was transformed into *E. coli* MG1655, resulting in the construction of a green fluorescent strain (MG1655-*gfp*). HvKP and MG1655-*gfp* were secondarily enriched to the logarithmic growth phase. After washing and resuspending with PBS, hvKP and MG1655-*gfp* were mixed in a 1:1 amount. Thirty microliters of the mixture was added to 3 mL LB medium and incubated at 37°C with shaking at 200 rpm for 24 h. Then, the mixture was diluted to an appropriate concentration, and 20 µL was spread onto LB agar plates. After being cultured for 12 h, CFUs of hvKP without fluorescence and MG1655-*gfp* with green fluorescence were calculated.

### Real-time quantitative polymerase chain reaction (RT-qPCR)

RT-qPCR was performed in the QuantStudio Real-Time PCR Software (ViiA7 Real-Time PCR Systems). Total RNA was extracted using *AG RNAex Pro* Reagent (Accurate Biology, AG21102). Then, genomic DNA elimination and cDNA synthesis were performed using *Evo M-MLV* RT Mix Kit (Accurate Biology, AG11728). RT-qPCR was carried out in a total volume of 10 µL in a 384-well optical reaction plate containing 5 µL of 2× SYBR Green *Pro Taq* HS Premix II (Accurate Biology, AG11719), 0.75 µL of cDNA, and two target gene specific primers (final concentration: 0.2 µM). 23S rRNA was used as an endogenous reference control for standardization, and the relative differences in mRNA expression were calculated using 2^−ΔΔCt^. Primers used for RT-qPCR are listed in Table S2.

### β-Galactosidase assays

The transcriptional reporter plasmid pME6522, carrying the *lacZ* gene (encoding β-galactosidase), was utilized for promoter activity analysis. The T6SS-1 promoter (PT6SS-1) and *mrkH* promoter (*PmrkH*) were PCR-amplified and cloned into the EcoRI/BstI sites upstream of the *lacZ* gene, generating pMEO-PT6SS-1 and pMEO-*PmrkH* constructs. All plasmids were verified by Sanger sequencing (Sangon Biotech) before transformation into ATCC43816 WT or Δ*dcuS* mutant. For the β-galactosidase activity measurements, bacterial cultures were grown at 37°C to mid-log phase. The β-galactosidase activity was quantified using the Miller method (80).

### ATP detection

ATP Assay Kit (Beyotime, S0026) was used to detect the bacterial intracellular ATP level. According to the manufacturer’s instructions, logarithmic bacterial strains were resuspended by PBS to an optical density of 0.8 at 600 nm, then 2 mL of suspended bacterial culture was added into a new Eppendorf tube. After centrifugation, the supernatant was discarded, and 2 mL lysate was added to the lysed bacterial sediment. The bacterial lysate was heated to 100°C for 15 min to completely lyse. After complete lysis, bacteria were centrifuged at 1,200 × *g* for 5 min at 4°C, and the supernatant was taken for subsequent assays. ATP detection was performed in a black 96-well plate. Twenty microliters lysate supernatant was added into 100 µL ATP detection working liquid. After completely mixing, fluorescence intensity was tested by microplate reader (Tecan, Infinite M1000 PRO). According to fluorescence standard curve, ATP concentration was calculated.

### Statistical analysis

Statistical analyses were performed using Graph Prism 9 (GraphPad Software, San Diego, CA). For normal data with homogeneity of variance, Student’s *t*-test was used for comparison of two groups. One-way analysis of variance (ANOVA) or two-way ANOVA was used for comparison when more than two groups were compared. The log-rank test was used to compare survival rates. *P* < 0.05 was considered statistically significant (*P < 0.05, **P < 0.01, ***P < 0.001, ****P < 0.0001).

## SUPPLEMENTAL MATERIAL

Supplemental figures spectrum.01453-25-s0001.pdf Figures S1 to S9.

Supplemental tables spectrum.01453-25-s0002.docx Tables S1 and S2.

ASM does not own the copyrights to Supplemental Material that may be linked to, or accessed through, an article. The authors have granted ASM a non-exclusive, world-wide license to publish the Supplemental Material files. Please contact the corresponding author directly for reuse.
