# Supplementary material for: Succinate-driven virulence enhancement in hypervirulent Klebsiella pneumoniae via DcuSR two-component system
Source: Microbiol Spectr. 2025 Nov 13;14(1):e01453-25. doi: 10.1128/spectrum.01453-25 (PMC12772337; doi:10.1128/spectrum.01453-25)
Supplement: Supplemental figures — Figures S1 to S9. [file spectrum.01453-25-s0001.pdf]

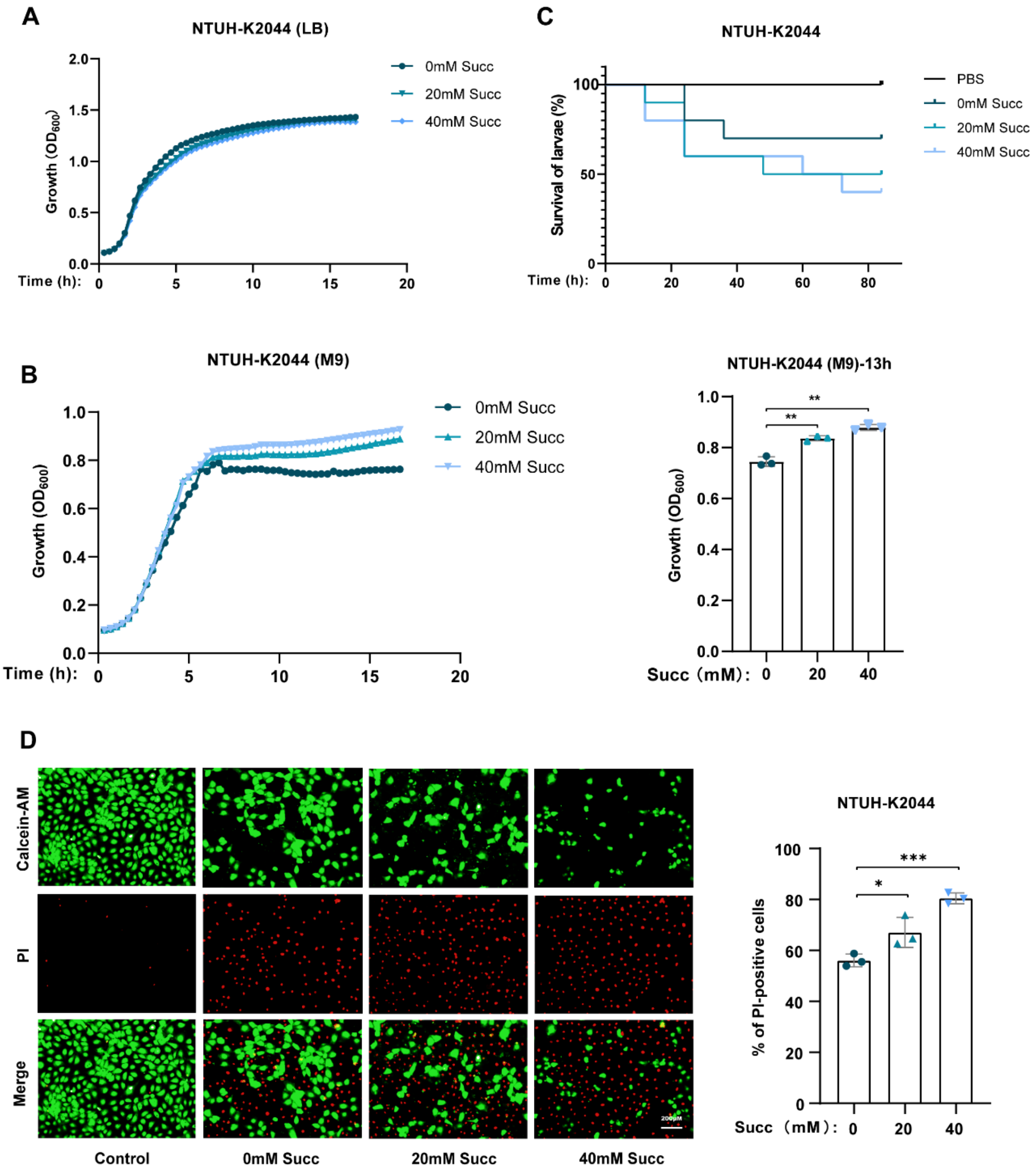

FIG S1 Succinate enhances the virulence and pathogenicity of NTUH-K2044. Growth curve of NTUH-K2044 in LB (A) or M9 medium (B) supplemented with 0, 20, 40 mM succinate. (C) Toxicity assay of *Galleria mellonella* larvae infected with NTUH-K2044 pretreated with 0, 20, 40 mM succinate. Each larva was injected with equal amounts of NTUH-K2044 pretreated with different concentrations of succinate. (D) Cytotoxicity assay of IEC6 intestinal epithelial cells infected with NTUH-K2044 pretreated with 0, 20, 40 mM succinate. Living cell was stained with calcein-AM (green), and the dead cell was stained with propidium iodide (red). Original magnification, 10×. Scale bar, 200 μm. The cytotoxicity assay results were quantified based on the mortality percentage of IEC6 cells after infected by NTUH-K2044 (MOI=100) for 12h. Succ, Succinate; PI, propidium iodide. All data are expressed as mean ±SD of three independent biological replicates. (n=3). **P* < 0.05, ***P* < 0.01, ****P* < 0.001.

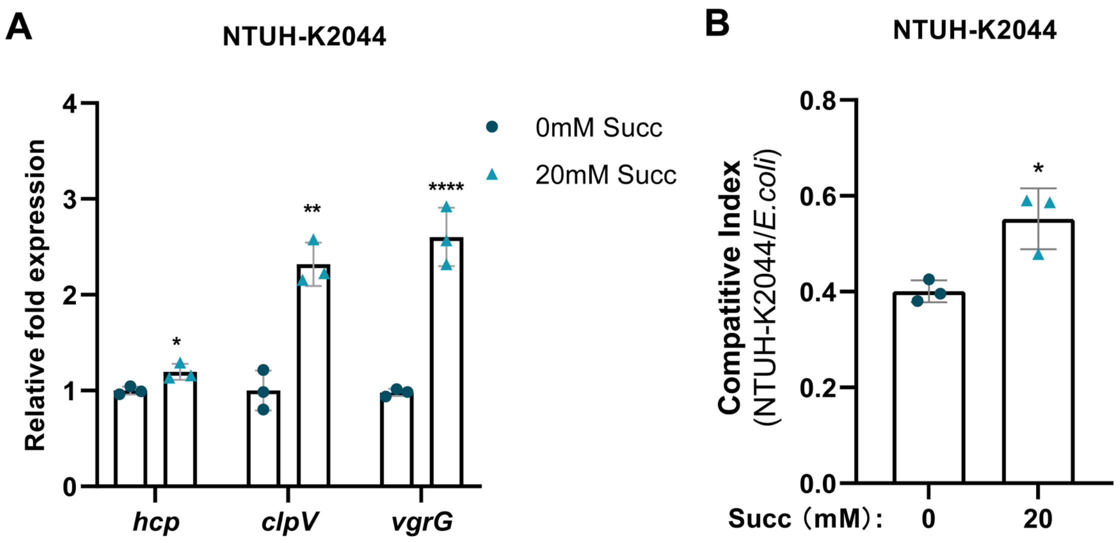

FIG S2 Succinate facilitates NTUH-K2044 T6SS genes expression. (A) RT-qPCR to determine T6SS genes, including *hcp*, *clpV* and *vgrG*, expression changes in NTUH-K2044 in LB supplemented with 0, 20mM succinate. (B) Competitive index (CI) analysis of NTUH-K2044 against *E. coli* (MG1655) at a 1:1 ratio in LB broth with 0, 20 mM succinate for 24 h. Succ, Succinate. Data are expressed as mean ±SD of three independent biological replicates. *P < 0.05, **P < 0.01, ****P < 0.0001.

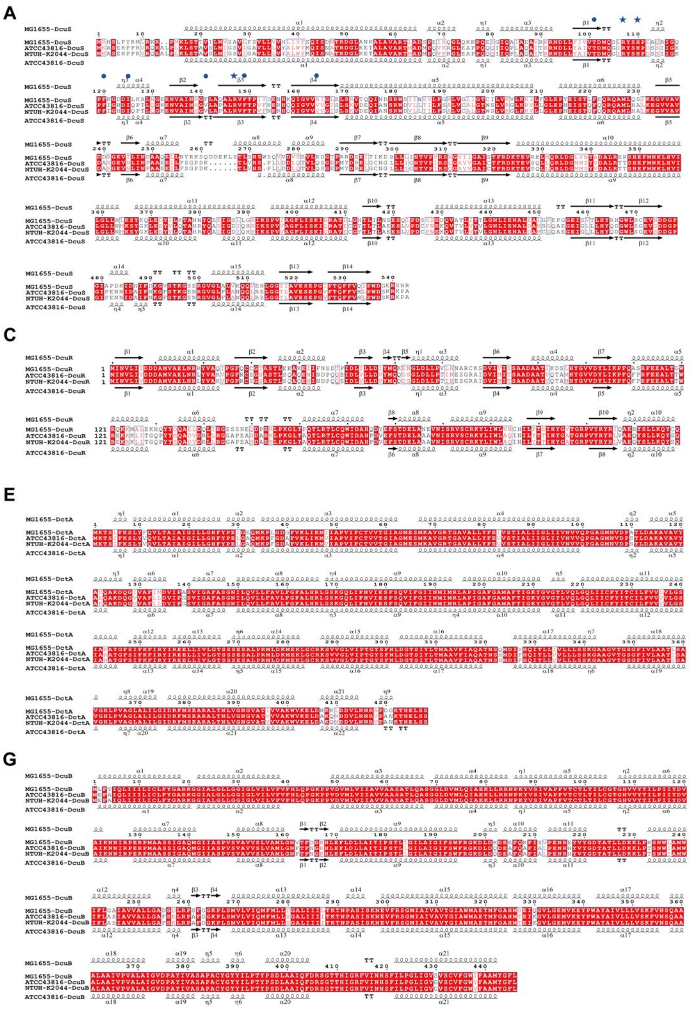

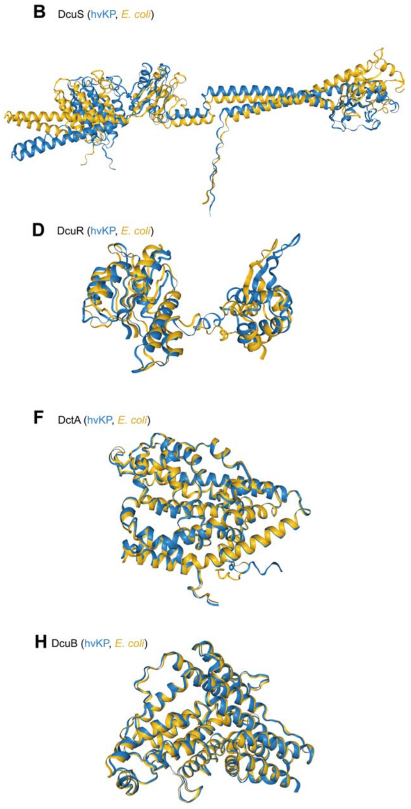

I

Protein	Identity (%)	Similarity (%)	TM Score
DcuS	56.33	90.15	0.56
DcuR	66.95	93.71	0.80
DctA	93.69	99.07	0.98
DcuB	94.17	98.73	0.99

FIG S3 Sequence alignment of proteins between hvKP and *E. coli*. Amino acid sequences of DcuS, DcuR, DctA and DcuB were retrieved from the National Center Biotechnology Information (NCBI)(1), and the corresponding structural files (PDB) were obtained from the Uniport database(2). Amino acid and secondary structure alignment of DcuS (A), DcuR (C), DctA (E) and DcuB (G) between hvKP (ATCC43816/NTUH-K2044) and *E. coli* MG1655. The amino acid sequences were aligned and visualized using Clustal Omega(3) and ESPript3.0(4) respectively. White letters in red boxes refer to strict identity, and red letters in white boxes indicate similarity. Universally conserved residues within the binding pocket between DcuS and C_4-DC were marked with blue asterisks, while subtype-specific residues were annotated with blue circles. 3D Structural alignment of DcuS (B), DcuR (D), DctA (F) and DcuB (H) between hvKP (ATCC43816/NTUH-K2044, blue) and *E. coli* MG1655 (yellow), generated using the FoldSeek(5). (I) Sequence and structural conservation metrics of DcuS, DcuR, DctA and DcuB between hvKP (ATCC43816/NTUH-K2044) and *E. coli* MG1655. Identity, percentage of identical amino acids in pairwise alignment; Similarity, percentage of conserved biochemical properties; TM-Score, Global structural similarity metric (0-1 scale, where > 0.5 indicates similar folds) (6).

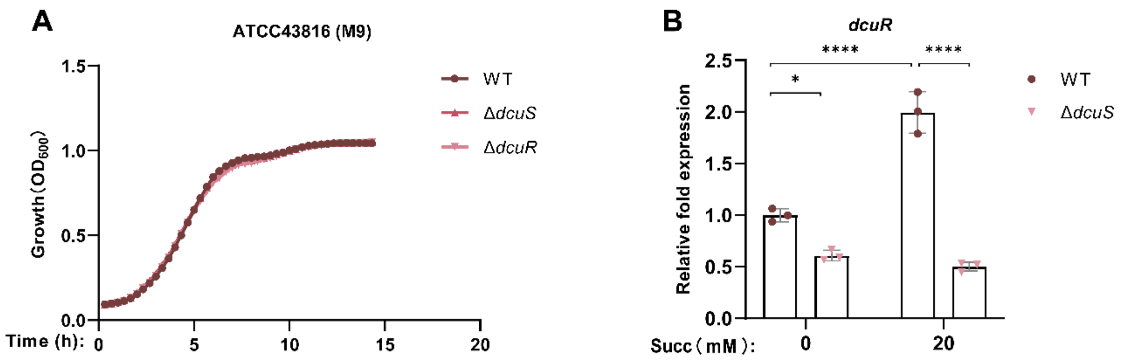

FIG S4 DcuS is required for succinate-induced *dcuR* activation in ATCC43816. (A) Growth curve of ATCC43816 WT, Δ*dcuS* and Δ*dcuR* at 37°C in M9 medium. (B) RT-qCR to determine *dcuR* expression changes of ATCC43816 WT and Δ*dcuS* grown in LB medium supplemented 0 or 20mM succinate. Succ, succinate; ATCC43816 WT, ATCC43816 wild-type strain; Δ*dcuS*, *dcuS* knock-out mutant of ATCC43816. All data are expressed as mean ±SD of three independent biological replicates. (n=3). *$P < 0.05$, ****$P < 0.0001$.

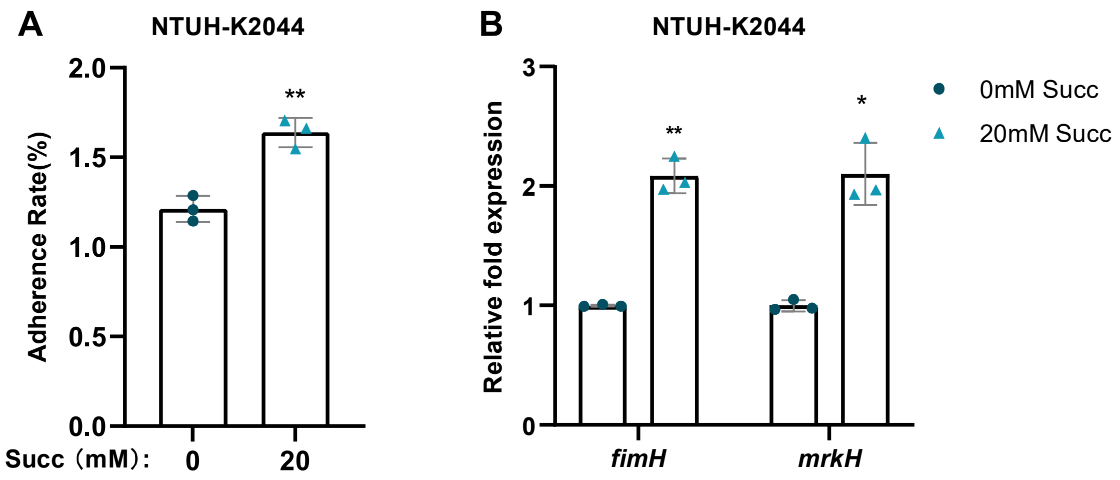

FIG S5 Succinate enhances NTUH-K2044 adhesion ability. (A) Adherence rate of NTUH-K2044 to NCM460 intestinal epithelial cells. (B) RT-qPCR to determine *fimH*, *mrkH* expression changes in NTUH-K2044 grown in LB broth supplemented 0, 20mM succinate. Succ, Succinate. Data are presented as mean ±SD of three independent biological replicates. *$P < 0.05$, **$P < 0.01$.

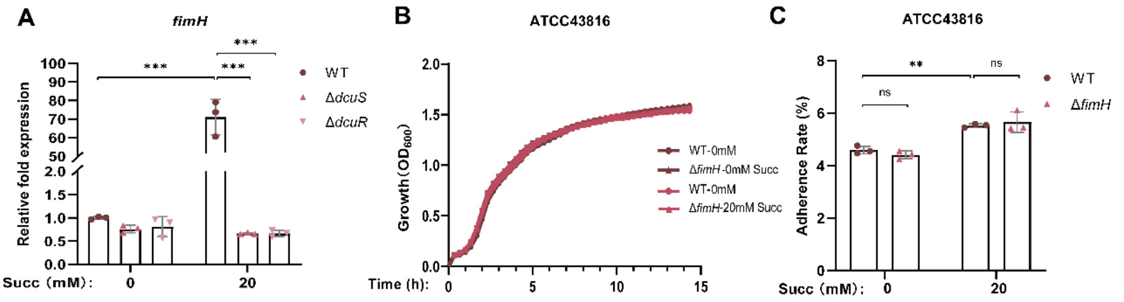

FIG S6 Type I fimbriae contribute minimally to succinate-enhanced adhesion in ATCC43816. (A) Fold expression changes of *fimH* gene of ATCC43816 WT, Δ*dcuS* and Δ*dcuR* in LB medium treated with or without 20 mM succinate. (B) Growth curves of ATCC43816 WT, Δ*fimH* at 37°C in LB medium with 0, 20 mM succinate. (C) Adherence rate of ATCC43816 WT, Δ*fimH* to NCM460 cells. Succ, succinate; ATCC43816 WT, ATCC43816 wild-type strain; Δ*fimH*, *fimH* knock-out mutant of ATCC43816. All data are presented as mean ± SD of three independent biological replicates. ns, not significant, $^{**}P < 0.01$, $^{***}P < 0.001$.

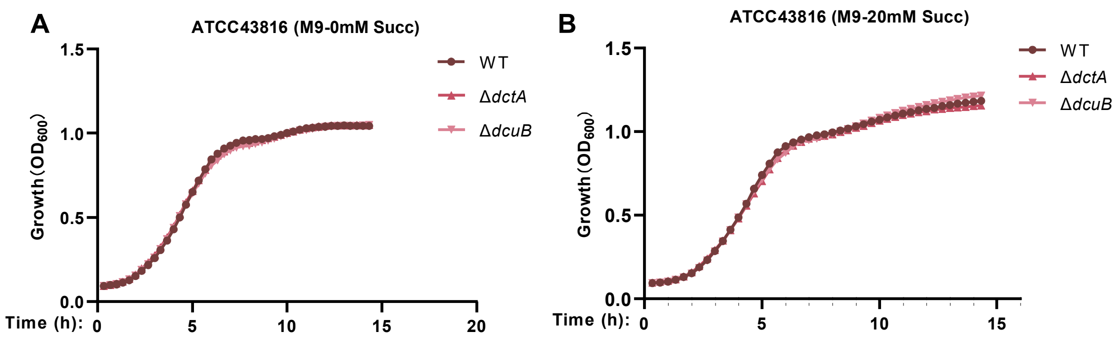

FIG S7 Growth curves of ATCC43816 WT, Δ*dctA* and Δ*dcuB* in M9 medium. (A) Growth curves of ATCC43816 WT, Δ*dctA* and Δ*dcuB* at 37°C in M9 medium added 0 mM succinate. (B) Growth curves of ATCC43816 WT, Δ*dctA* and Δ*dcuB* at 37°C in M9 medium added 20 mM succinate. Succ, succinate; ATCC43816 WT, ATCC43816 wild-type strain; Δ*dctA*, *dctA* knock-out mutant of ATCC43816; Δ*dcuB*, *dcuB* knock-out mutant of ATCC43816. Data are presented as mean ±SD of three independent biological replicates.

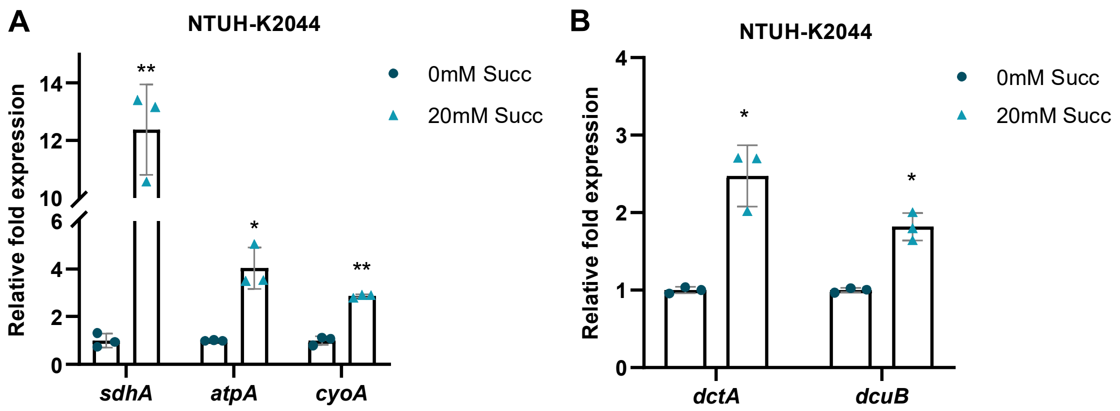

FIG S8 Impact of succinate on the TCA cycle in NTUH-K2044. (A) RT-qPCR was performed to measure the expression of *sdhA*, *atpA*, *cyoA* in NTUH-K2044. (B) RT-qPCR was performed to measure the expression of succinate transporters coding genes *dctA* and *dcuB* in NTUH-K2044. Succ, succinate. Data are presented as mean ± SD of three independent biological replicates. **P* < 0.05, ***P* < 0.01.

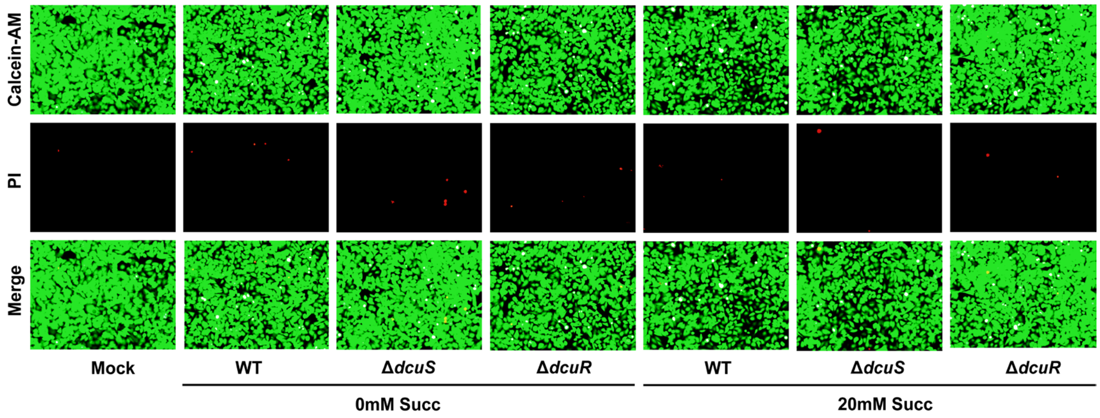

FIG S9 Negligible cell damage in NCM460 cells following 3-hour infection with ATCC43816. (A) Cytotoxicity assay of NCM460 intestinal epithelial cells infected with ATCC43816 WT, Δ*dcuS*, or Δ*dcuR* pretreated with 0, 20 mM succinate for 3 hour. Living cell was stained with calcein-AM (green), and the dead cell was stained with propidium iodide (red).

SUPPLEMENTAL REFERENCE

1. Sayers EW, Beck J, Bolton EE, Brister JR, Chan J, Connor R, Feldgarden M, Fine AM, Funk K, Hoffman J, Kannan S, Kelly C, Klimke W, Kim S, Lathrop S, Marchler-Bauer A, Murphy TD, O'Sullivan C, Schmieder E, Skripchenko Y, Stine A, Thibaud-Nissen F, Wang J, Ye J, Zellers E, Schneider VA, Pruitt KD. 2024. Database resources of the National Center for Biotechnology Information in 2025. Nucleic Acids Res 53: D20–D29.

2. UniProt Consortium. 2025. UniProt: the Universal Protein Knowledgebase in 2025. Nucleic Acids Res 53: D609–D617.

3. Sievers F, Wilm A, Dineen D, Gibson TJ, Karplus K, Li W, Lopez R, McWilliam H, Remmert M, Söding J, Thompson JD, Higgins DG. 2011. Fast, scalable generation of high-quality protein multiple sequence alignments using Clustal Omega. Mol Syst Biol 7:539.

4. Robert X, Gouet P. 2014. Deciphering key features in protein structures with the new ENDscript server. Nucleic Acids Res 42: W320–W324.

5. van Kempen M, Kim SS, Tumescheit C, Mirdita M, Lee J, Gilchrist CLM, Söding J, Steinegger M. 2024. Fast and accurate protein structure search with Foldseek. Nat Biotechnol 42:243–246.

6. Zhang, Y., & Skolnick, J. 2004. Scoring function for automated assessment of protein structure template quality. Proteins 57(4), 702–710.
