# Supplementary material for: Succinate-driven virulence enhancement in hypervirulent Klebsiella pneumoniae via DcuSR two-component system
Source: Microbiol Spectr. 2025 Nov 13;14(1):e01453-25. doi: 10.1128/spectrum.01453-25 (PMC12772337; doi:10.1128/spectrum.01453-25)
Supplement: Supplemental tables — Tables S1 and S2. [file spectrum.01453-25-s0002.docx]

**TABLE S1** Bacterial strains and plasmids used in this study

| **Strains/Plasmids** | **Characteristics** | **Source** |
| --- | --- | --- |
| ***Klebsiella pneumoniae*** | | |
| NTUH-K2044 | wild-type strain, hyper-virulent *Klebsiella pneumoniae* (K1) | Lab collection |
| ATCC43816 | wild-type strain, hyper-virulent *Klebsiella pneumoniae* (K2) | Lab collection |
| ATCC43816 Δ*dcuS* | *dcuS* deletion mutant in ATCC43816, Apr^S^, Hyg^S^ | This work |
| ATCC43816 Δ*dcuR* | *dcuR* deletion mutant in ATCC43816, Apr^S^, Hyg^S^ | This work |
| ATCC43816 Δ*dctA* | *dctA* deletion mutant in ATCC43816, Apr^S^, Hyg^S^ | This work |
| ATCC43816 Δ*dcuB* | *dcuB* deletion mutant in ATCC43816, Apr^S^, Hyg^S^ | This work |
| ATCC43816 Δ*fimH* | *fimH* deletion mutant in ATCC43816, Apr^S^, Hyg^S^ | This work |
| ATCC43816 Δ*mrkH* | *mrkH* deletion mutant in ATCC43816, Apr^S^, Hyg^S^ | This work |
| ATCC43816 Δ*clpV* | *clpV* deletion mutant in ATCC43816, Apr^S^, Hyg^S^ | This work |
| ATCC43816Δ*dcuR*Δ*clpV* | *dcuR/clpV double* mutant in ATCC43816, Apr^S^, Hyg^S^ | This work |
| ATCC43816Δ*dcuR*Δ*clpV*(p*dcuR*) | *dcuR-*complemented strain of *dcuR/clpV* double mutant, Apr^R^, Hyg^S^ | This work |
| ATCC43816Δ*dcuR*Δ*clpV*(p*clpV*) | *clpV-*complemented strain of *dcuR/clpV* double mutant, Apr^R^, Hyg^S^ | This work |
| ATCC43816Δ*dcuR*Δ*mrkH* | *dcuR/mrkH double* mutant in ATCC43816, Apr^S^, Hyg^S^ | This work |
| ATCC43816Δ*dcuR*Δ*mrkH*(p*dcuR*) | *dcuR-*complemented strain of *dcuR/mrkH* double mutant, Apr^R^, Hyg^S^ | This work |
| ATCC43816Δ*dcuR*Δ*mrkH*(p*mrkH*) | *mrkH-*complemented strain of *dcuR/mrkH* double mutant, Apr^R^, Hyg^S^ | This work |

**Continued TABLE S1** Bacterial strains and plasmids used in this study

| **Strains/Plasmids** | **Characteristics** | **Source** |
| --- | --- | --- |
| ATCC43816(PT6SS-1-*lacZ*) | ATCC43816 derivative containing the pMEO-PT6SS-1 plasmid | This work |
| ATCC43816Δ*dcuR*(PT6SS-1-*lacZ*) | ATCC43816Δ*dcuR* derivative containing the pMEO-PT6SS-1 plasmid | This work |
| ATCC43816(*PmrkH*-1-*lacZ*) | ATCC43816 derivative containing the pMEO-*PmrkH* plasmid | This work |
| ATCC43816Δ*dcuR*(*PmrkH*-1-*lacZ*) | ATCC43816Δ*dcuR* derivative containing the pMEO-*PmrkH* plasmid | This work |
| ***Escherichia coli*** |  |  |
| MG1655 | wild-type strain, *E. coli* K-12, a laboratory strain that colonizes the lower gut of animals | Lab collection |
| MG1655-*gfp* | MG655 containing a *gfp-*expression plasmid | Lab collection |
| **Plamids** |  |  |
| pACBSR-Hyg | containing the λ-Red system, controlled by an arabinose-inducible promoter, temperature sensitive, Hyg^R^ | ^[1]^ |
| pIJ773 | containing FRT sites, Apr^R^ | ^[1]^ |
| pFLP-Hyg | containing the FLP recombinase, temperature sensitive, Hyg^R^ | ^[1]^ |
| pSTVA | containing P*lac* promoter, derived from pSTV28, Apr^R^ | Lab collection |
| pSTVA-*clpV* | *clpV* overexpression plasmid, derived from pSTVA, Apr^R^ | This work |
| pSTVA-*mrkH* | *mrkH* overexpression plasmid, derived from pSTVA, Apr^R^ | This work |
| pME6522 | Promter-less *lacZ* reporter plasmid, Tc^R^ | Lab collection |

**Continued TABLE S1** Bacterial strains and plasmids used in this study

| **Strains/Plasmids** | **Characteristics** | **Source** |
| --- | --- | --- |
| pMEO-PT6SS-1 | containing the T6SS-1 promoter upstream of *lacZ* gene, lacing *lacI* binding site, derived from pME6522 | This work |
| pMEO-*PmrkH* | containing the T6SS-1 promoter upstream of *lacZ* gene, lacing *lacI* binding site, derived from pME6522 | This work |

**References:**

[1] He J, Shi Q, Chen Z, et al. Opposite evolution of pathogenicity driven by in vivo wzc and wcaJ mutations in ST11-KL64 carbapenem-resistant *Klebsiella pneumoniae*[J]. Drug Resistance Updates: Reviews and Commentaries In Antimicrobial and Anticancer Chemotherapy, 2023, 66: 100891.

**TABLE S2** Sequences of cloning primers and RT-qPCR primers

| **Name** | **Purpose** | **Sequence （5'→3'）** |
| --- | --- | --- |
| *dcuS*-KO-F | λ-Red konck-out of *dcuS* | CTTTATGTATGCCGCGCTATCGGCGATAATAATCTTTCACTGAATCTACCCGGGGTCGTCATTCCGGGGATCCGTCGACC |
| *dcuS*-KO-R | λ-Red konck-out of *dcuS* | GAGGATCTCTTCCGCCGCGGGGTTGATGAGATTTACCTCGCCATGGGCATCGACCGCCACTGTAGGCTGGAGCTGCTTC |
| *dcuR*-KO-F | λ-Red konck-out of *dcuR* | CGTATTTACCCAATTTTTTGTTCATCTCCCCTGGGATAGTAAAAGGAAACCTGCGTGATAATTCCGGGGATCCGTCGACC |
| *dcuR*-KO-R | λ-Red konck-out of *dcuR* | CGGCTGCGTTTTGCGCCGCTGGACAGCGGCTATCGGGTTATTGACTGTACTGCTTAAGCATGTAGGCTGGAGCTGCTTC |
| *dctA*-KO-F | λ-Red konck-out of *dctA* | TTTAAGCCACAGACGTCAAATATCCCTTCTTGCTCTGTGGTTATTCCCAAAGGACACCCTATTCCGGGGATCCGTCGACC |
| *dctA*-KO-R | λ-Red konck-out of *dctA* | CCGAAGATAACCTGGGAGAAGCTTTCAATGACGTTGAAAATCAGCTGGCCCTTGCTGCCCTGTAGGCTGGAGCTGCTTC |
| *dcuB*-KO-F | λ-Red konck-out of *dcuB* | AATCCCGAGAGGCAGGTAGGGATCATTTTTAGATCTGAGTGGACAGGCTCTGGGGGCTGCATTCCGGGGATCCGTCGACC |
| *dcuB*-KO-R | λ-Red konck-out of *dcuB* | ATAGACGTAATGGCGGTTCTCCGGGAGCGCAATAAACGCCTGGAAGGCTTCGTCCTGATCTGTAGGCTGGAGCTGCTTC |
| *clpV*-KO-F | λ-Red konck-out of *clpV* | AACACAATCTGTCTGCCTGACCTTGTGCGCTTGGGGTTCCTGTTCAGGAATAGCGACGGGATTCCGGGGATCCGTCGACC |
| *clpV*-KO-R | λ-Red konck-out of *clpV* | TTTGACCCCGGCGCCGGCCTGCAGCAGACCGAGGTCAAGGGTGCGCACCGACACCGGCTTTGTAGGCTGGAGCTGCTTC |
| *fimH*-KO-F | λ-Red konck-out of *fimH* | CGGCGCCACGCAAGGCACCATTCAGGCGGTGATTAACGTCACCTATACCTACGCCTGAGCATTCCGGGGATCCGTCGACC |

**Continued TABLE S2** Sequences of cloning primers and RT-qPCR primers

| **Name** | **Purpose** | **Sequence （5'→3'）** |
| --- | --- | --- |
| *fimH-*KO-R | λ-Red konck-out of *fimH* | CAGCTGCTGGCTCTGCGCGCAGTGGACGGTGAGCGGCACGGCCATCGATCCCGGGTAGTCTGTAGGCTGGAGCTGCTTC |
| *mrkH*-KO-F | λ-Red konck-out of *mrkH* | ACATCAAACGCTCATAAAAACATCTGATTAACCCAATCAGAATGTTGCTATTGCTATAAGATTCCGGGGATCCGTCGACC |
| *mrkH*-KO-R | λ-Red konck-out of *mrkH* | GATAGATTGAGTGACCAATGAGATTGTCATTGGTGTACAGCAATATACTGTCCAAGGTTGTGTAGGCTGGAGCTGCTTC |
| check-*dcuS*-F | Confirm the knock-out of *dcuS* | GCCAGTTCCATAGTGTCTCCCTTA |
| check-*dcuS*-R | Confirm the knock-out of *dcuS* | GCTCATCATGCCGTCCAGTC |
| check-*dcuR*-F | Confirm the knock-out of *dcuR* | AATCGGAACCCGGCGTATTT |
| check-*dcuR*-R | Confirm the knock-out of *dcuR* | ACAACGAACTCGACAGCGTGAC |
| check-*dcuB*-F | Confirm the knock-out of *dcuB* | GGGATTACTTCTGTTGCTGTATCTTCG |
| check-*dcuB*-R | Confirm the knock-out of *dcuB* | GGCGACCGACATGATTGAACTCT |
| check-*dctA*-F | Confirm the knock-out of *dctA* | CGTTGCACTTTCGGCGTTAGTTAT |
| check-*dctA*-R | Confirm the knock-out of *dctA* | AATACCTGCCACGACAAACCATTC |
| check-*clpV*-F | Confirm the knock-out of *clpV* | CCTGACCTTGTGCGCTTGGG |
| check-*clpV*-R | Confirm the knock-out of *clpV* | GCTGCTCGAACTCGCCTTTG |
| check-*fimH*-F | Confirm the knock-out of *fimH* | CAGTCGGTACAGGTGGATGAGG |
| check-*fimH*-R | Confirm the knock-out of *fimH* | CGGAGGCGGTATTGGTGAAG |
| check-*mrkH*-F | Confirm the knock-out of *mrkH* | GAACCCTATCCCTCCTCAAT |
| check-*mrkH*-R | Confirm the knock-out of *mrkH* | TGGATGACATCGGCATAACT |
| INA-*tssH*-F | Construction of *tssH* overexpression plasmid | ATGACCATGATTACGAATTCAAACAATAAGAGAGGATCTC |
| INA-*tssH*-R | Construction of *tssH* overexpression plasmid | CGGGTACCGAGCTCGAATTCACGCTACTCATCGGATATTG |
| INA-*mrkH*-F | Construction of *mrkH* overexpression plasmid | ATGACCATGATTACGAATTCACTATTTACAAGGGATGCAT |
| INA-*mrkH*-R | Construction of *mrkH* overexpression plasmid | CGGGTACCGAGCTCGAATTCGTACAGCAATATACTGTCCA |
| InA-*dcuR*-F | Construction of *dcuR* overexpression plasmid | ATGACCATGATTACGAATTCCGTGATAAATGTTTTAATTG |
| InA-*dcuR*-R | Construction of *dcuR* overexpression plasmid | CGGGTACCGAGCTCGAATTCGTTATTGACTGTACTGCTTA |

**Continued TABLE S2** Sequences of cloning primers and RT-qPCR primers

| **Name** | | **Purpose** | | **Sequence （5'→3'）** |
| --- | --- | --- | --- | --- |
| pME6-F | | Construction of PT6SS-1-*lacZ* | | AATGGCAAAAGCTTCGAATTCCTTTGTGTCTCGTTGTGGGC |
| pME6- R | | Construction of PT6SS-1-*lacZ* | | TAGCTGTTTCCTGTGTGAAATTCTGCAGAGCAGTTCCTTTCCTTAAGG |
| pME-*mrkH*-F | | Construction of *PmrkH*-*lacZ* | | AATGGCAAAAGCTTCGAATTCCCTGCCAGACAAAATGGAGG |
| pME-*mrkH* -R | | Construction of *PmrkH*-*lacZ* | | TAGCTGTTTCCTGTGTGAAATTCTGCAGATGCATCCCTTGTAAATAGT |
| q-*dcuS*-F | | RT-qPCR | | CAGAGCGGCGAGCCAATGTA |
| q-*dcuS*-R | | RT-qPCR | | GCTGCGAATGGGCACTGTAT |
| q-*dcuR*-F | | RT-qPCR | | GCCGAACTGAATCGCCTGTA |
| q-*dcuR*-R | RT-qPCR | GAGATCGAGCCCGCTGTCCT | | |
| q-*dctA*-F | RT-qPCR | GGAAGAGCTGCTGATTGTGC | | |
| q-*dctA*-R | RT-qPCR | CCTGGGCGATAAAGACTGC | | |
| q-*dcuB*-F | RT-qPCR | TTAAAGGCGTGCTTGGTGAG | | |
| q-*dcuB*-R | RT-qPCR | CCAGATCGCTTGGGTAGGTC | | |
| q-*fimH-*F | RT-qPCR | CCAGCCGTCCTGTATCTGAC | | |
| q-*fimH-*R | RT-qPCR | GGGACCACCACGTCGTTATT | | |
| q-*mrkH*-F | RT-qPCR | CGAGATCACCATCGACCTGG | | |
| q-*mrkH-*R | RT-qPCR | GTCATCGAGATGGCGGAACT | | |
| q-*hcp-*F | RT-qPCR | CAAAAACCCCGCCTACGA | | |
| q-*hcp-*R | RT-qPCR | TCGTAGCGCAGCTCAATCTG | | |
| q-*vgrG*-F | RT-qPCR | ATGAGTAGCGTGAAATCGTTG | | |
| q-*vgrG*-R | RT-qPCR | CAATACGGTAGCGGAACG | | |
| q-*clpV-*F | RT-qPCR | CCTGAAGTCCCGCTATGCC | | |
| q-*clpV-*R | RT-qPCR | CGAGTAAATCCACTGCCTTGTC | | |
| RT-*sdhA*-5F | RT-qPCR | GACCGCCGTATCCGCTAACTTC | | |
| RT-*sdhA*-3R | RT-qPCR | TGGATTCCGACTCTGGCAGGTA | | |
| RT-*atpA*-5F | RT-qPCR | CGTGGTTACCTGGCTGATGTAGA | | |
| RT-*atpA*-3R | RT-qPCR | AGGACTGGGTTGCTTTGAAGGAA | | |
| RT-*cyoA*-5F | RT-qPCR | GTCGTTATTCCCGCCGTCTTGA | | |
| RT-*cyoA*-3R | RT-qPCR | TTGGCTCCAGTGCGTGAGTG | | |

**Continued TABLE S2** Sequences of cloning primers and RT-qPCR primers

| **Name** | | **Purpose** | | | **Sequence （5'→3'）** |
| --- | --- | --- | --- | --- | --- |
| q-*mrkA*-F | RT-qPCR | | | CTAACTGCCAGGCTGCTGAT | |
| q-*mrkA*-R | RT-qPCR | | | ACCAGCTGGATGTTCTGAGC | |
| q-*mrkB*-F | RT-qPCR | | | TAAAGAGACGCTGTGGTGGC | |
| q-*mrkB*-R | | | RT-qPCR | CAGTCCGGCAGGACGATAAA | |
| 23s-F | | | RT-qPCR | GGTAGGGGAGCGTTCTGTAA | |
| 23s-R | | | RT-qPCR | TCAGCATTCGCACTTCTGAT | |
